# Effects of host migration and travel loss on strain competition in a two-patch SIR model

**DOI:** 10.1007/s00285-026-02397-z

**Published:** 2026-04-17

**Authors:** Bo-Sheng Chen, Chang-Hong Wu

**Affiliations:** 1 Department of Applied Mathematics, National Yang Ming Chiao Tung University, Hsinchu, 300 Taiwan; 2 National Center for Theoretical Sciences, Taipei, 106 Taiwan

**Keywords:** Two-strain SIR model, Migration, stability, Competitive exclusion, 34D23, 92D30

## Abstract

This paper investigates how host migration with travel loss affects competition between two pathogen strains in animal populations using a two-patch SIR model. We establish sufficient conditions involving migration rates and travel losses that ensure the global asymptotic stability of a single-strain endemic equilibrium or a coexistence equilibrium. Complemented by numerical simulations, our results provide insights into how competitive outcomes are shaped by the interplay among migration asymmetry, travel loss, and epidemiological traits.

## Introduction

The dispersal of species plays a crucial role in the spread of diseases. Migratory animals, such as birds, mammals, and insects, have been associated with the geographic spread of diseases like avian influenza, West Nile virus, and Ebola. Migration may not always increase the risk of pathogen transmission. For example, it can reduce the spread of diseases through mechanisms such as “migratory escape,” where animals avoid infection by leaving contaminated habitats, or “migratory culling,” where infected animals may die during long-distance migration due to their inability to complete the journey, thereby reducing the pathogen prevalence in the population (see Altizer et al. (2011)).

In spatially heterogeneous environments, host movement can also affect which strain eventually dominates. Experimental studies suggest that restricting host movement may shift strain dominance toward pathogens with lower infectivity and/or lower virulence (see Boots and Mealor (2007), Eshelman et al. (2010)). These phenomena indicate that migration is not only a strategy for animals to adapt to their environment, but also a factor that influences disease dynamics and transmission patterns.

Movement typically imposes a cost on the population, often requiring energy expenditure and increasing the risk of predation or mortality from environmental exposure. Incorporating travel loss provides a natural way to quantify such movement costs and understand the impact on population movement patterns and spatial distributions (see DeAngelis et al. (2011), Gibbs et al. (2010), Yoder et al. (2004)). Given this complexity, it is essential to understand how migration, particularly involving travel loss, affects the dynamics of pathogens in animal populations. In particular, we may ask: in the presence of travel loss, how does host migration determine whether one strain excludes the other or whether coexistence is possible?

To address this, we consider a two-patch, two-strain SIR model with travel loss and class-dependent, asymmetric migration, aiming to investigate how host movement shapes the outcome between competitive exclusion and coexistence. We assume that there is a certain probability $\varepsilon _{ij}\in [0,1)$ of individuals dying during the migration process from the *j*-th patch to the *i*-th patch (DeAngelis et al. 2011). However, due to factors such as the increased risk of predation or mortality during the migration of infected individuals or culling policies for disease prevention, the travel loss of infected individuals may be different from that of susceptibles. Thus, it is plausible to assume that $\varepsilon _{ij}^S,{\varepsilon _{ij}^{I},\varepsilon _{ij}^J},\varepsilon _{ij}^R\in [0,1)$ are travel losses of the susceptible class, the infectious classes of strains *I* and *J*, and the removed class, respectively, during the migration process from the *j*-th patch to the *i*-th patch.

To minimize mathematical complexity while capturing the essential impact of travel loss, we assume that the travel losses of the infectious classes are the same; that is,

$$\begin{aligned}\varepsilon _{ij}^I=\varepsilon _{ij}^J=:\varepsilon _{ij},\quad i,j\in \{1,2\},\quad i\ne j.\end{aligned}$$

We assume full cross-immunity, meaning that after infection by one strain, the host becomes completely immune to the other strain. Furthermore, we assume there are no co-infection or superinfection events. This leads us to consider the following ODE model:

1.1 $$\begin{aligned} {\left\{ \begin{array}{ll} S_1'=\Lambda _1-\beta ^I_1S_1I_1-\beta ^J_1S_1J_1-d_1S_1-m_{21}^SS_1+m_{12}^S(1-\varepsilon _{12}^S)S_2,\quad t\ge 0,\\ I_1'=\beta ^I_1S_1I_1-(d_1+\alpha _1^I+\gamma _1^I)I_1-m_{21}^II_1+m_{12}^I(1-\varepsilon _{12})I_2,\quad t\ge 0,\\ J_1'=\beta ^J_1S_1J_1-(d_1+\alpha _1^J+\gamma _1^J)J_1-m_{21}^JJ_1+m_{12}^J(1-\varepsilon _{12})J_2,\quad t\ge 0,\\ R_1'=\gamma _1^I I_1+\gamma _1^J J_1-d_1R_1-m_{21}^RR_1+m_{12}^R(1-\varepsilon _{12}^R)R_2,\quad t\ge 0,\\ S_2'=\Lambda _2-\beta ^I_2S_2I_2-\beta ^J_2S_2J_2-d_2S_2-m_{12}^SS_2+m_{21}^S(1-\varepsilon _{21}^S)S_1,\quad t\ge 0,\\ I_2'=\beta ^I_2S_2I_2-(d_2+\alpha _2^I+\gamma _2^I)I_2-m_{12}^II_2+m_{21}^I(1-\varepsilon _{21})I_1,\quad t\ge 0,\\ J_2'=\beta ^J_2S_2J_2-(d_2+\alpha _2^J+\gamma _2^J)J_2-m_{12}^JJ_2+m_{21}^J(1-\varepsilon _{21})J_1,\quad t\ge 0,\\ R_2'=\gamma _2^I I_2+\gamma _2^J J_2-d_2R_2-m_{12}^RR_2+m_{21}^R(1-\varepsilon _{21}^R)R_1,\quad t\ge 0, \end{array}\right. } \end{aligned}$$

with the initial data

1.2 $$\begin{aligned} S_i(0)>0,\quad I_i(0)>0,\quad J_i(0)>0,\quad R_i(0)\ge 0,\quad i=1,2, \end{aligned}$$

where $S_i(t),I_i(t),J_i(t)$ and $R_i(t)$ stand for the susceptible, strain-*I* infectious, strain-*J* infectious, and removed populations, respectively, at time *t* in the *i*-th patch ($i=1,2$). All parameters are nonnegative, and we assume that $\Lambda _i,d_i,\beta _i^k,\alpha _i^k,\gamma _i^k>0$ ($i=1,2,\ k=I,J$), $m_{ij}^{\ell }\ge 0$, $\ell \in \{S,I,J,R\}$, $i\ne j$, and the travel-loss parameters satisfy $0\le \varepsilon _{ij}^{S},\varepsilon _{ij},\varepsilon _{ij}^{R}<1$. The meaning of parameters are summarized in Table 1.

**Table 1:** Notation and parameter descriptions for system (1.1).

Symbol	Description
$\Lambda _i$	Recruitment rate into patch *i*.
$d_i$	Natural death rate in patch *i*.
$\beta _i^k$	Transmission rate of strain *k* ($k=I,J$) in patch *i*.
$\alpha _i^{k}$	Disease-induced death rate (or the virulence) of strain *k* ($k=I,J$) in patch *i*.
$\gamma _i^k$	Recovery rate of the strain-*k* infectious class ($k=I,J$) in patch *i*.
$m_{ij}^{S}$	Migration rate of susceptibles from patch *j* to patch *i*.
$m_{ij}^{k}$	Migration rate of strain-*k* infected hosts ($k=I,J$) from patch *j* to patch *i*.
$m_{ij}^{R}$	Migration rate of the removed class from patch *j* to patch *i*.
$\varepsilon _{ij}^{S}$	Fraction of susceptible migrants lost during travel from patch *j* to patch *i*.
$\varepsilon _{ij}$	Fraction of infected migrants lost during travel from patch *j* to patch *i*.
$\varepsilon _{ij}^{R}$	Fraction of removed migrants lost during travel from patch *j* to patch *i*.

The model allows asymmetric migration. Namely, for $\ell \in \{S,I,J,R\}$, we allow $m_{12}^{\ell }\ne m_{21}^{\ell }$. Since $R_1$ and $R_2$ do not appear in the equations satisfied by $S_i,I_i$ and $J_i$, the subsystem $(S_i,I_i,J_i)$ is independent of $(R_1,R_2)$. Therefore, we focus on the subsystem. Once $S_i(t)$, $I_i(t)$, and $J_i(t)$ are determined, each $R_i(t)$ can be recovered by solving its linear nonhomogeneous equation with forcing terms depending on $(S_i,I_i,J_i)$, subject to the initial data $R_i(0)\ge 0$.

Consequently, it suffices to focus our mathematical analysis on the equations satisfied by $S_i,I_i$ and $J_i$:

1.3 $$\begin{aligned} {\left\{ \begin{array}{ll} S_1'=\Lambda _1-\beta ^I_1S_1I_1-\beta ^J_1S_1J_1-d_1S_1-m_{21}^SS_1+m_{12}^S(1-\varepsilon _{12}^S)S_2,\quad t\ge 0,\\ I_1'=\beta ^I_1S_1I_1-(d_1+\alpha _1^I+\gamma _1^I)I_1-m_{21}^II_1+m_{12}^I(1-\varepsilon _{12})I_2,\quad t\ge 0,\\ J_1'=\beta ^J_1S_1J_1-(d_1+\alpha _1^J+\gamma _1^J)J_1-m_{21}^JJ_1+m_{12}^J(1-\varepsilon _{12})J_2,\quad t\ge 0,\\ S_2'=\Lambda _2-\beta ^I_2S_2I_2-\beta ^J_2S_2J_2-d_2S_2-m_{12}^SS_2+m_{21}^S(1-\varepsilon _{21}^S)S_1,\quad t\ge 0,\\ I_2'=\beta ^I_2S_2I_2-(d_2+\alpha _2^I+\gamma _2^I)I_2-m_{12}^II_2+m_{21}^I(1-\varepsilon _{21})I_1,\quad t\ge 0,\\ J_2'=\beta ^J_2S_2J_2-(d_2+\alpha _2^J+\gamma _2^J)J_2-m_{12}^JJ_2+m_{21}^J(1-\varepsilon _{21})J_1,\quad t\ge 0, \end{array}\right. } \end{aligned}$$

with the initial data

1.4 $$\begin{aligned} S_i(0)>0,\quad I_i(0)>0,\quad J_i(0)>0,\quad i=1,2, \end{aligned}$$

From a mathematical viewpoint, many theoretical results for spatial or metapopulation epidemic models have been established for single-pathogen (single-strain) systems. For example, Hethcote (1976) addressed an interesting question: Can a disease remain endemic by traveling geographically around a region? He considered a two-patch SIS model and showed that migration can sustain the endemicity of a disease in patches, even if the disease would eventually vanish in each isolated patch. Arino and Driessche (2003) proposed epidemic models involving *n* cities to examine the impact of inter-city travel on spatial transmission dynamics. When the assumption of symmetric migrations is removed, Wang and Zhao (2004) considered an *n*-patch SIS model and applied the uniform persistence theory to prove the existence of an endemic equilibrium. In the two-patch scenario, Wang (2004) and Brauer et al. (2008) considered a SIR model in which infectious individuals are unable to migrate. In Wang (2004), it was found that the disease can either go extinct (resp., prevail) in both patches or prevail in one patch and vanish in the other patch. The authors of Brauer et al. (2008) discovered that the movement of susceptible individuals can lead to the establishment of endemic disease. Using a graph-theoretical approach (Guo et al. 2008), Li and Shuai (2009) studied the global dynamics of an *n*-patch SIR model. With more emphasis on the effect of dispersal rates, Gao (2020) indicated that fast migration may decrease the basic reproduction number but increase the infection size in a multi-patch SIS model. For additional related works, we refer to Arino and Portet (2015); Chen et al. (2020); Jin and Wang (2005); Li and Peng (2019); Salmani and Driessche (2006) and the references cited therein.

In many infectious diseases, multiple pathogen strains can spread simultaneously and compete for the same host resources. One of the key principles of theoretical ecology is the competitive exclusion principle, which states that two species that compete for the same limited resource cannot coexist (see, e.g., Levin (1970)). In the single-patch and well-mixed settings, Bremermann and Thieme (1989) considered a modified Anderson and May model of host-parasite dynamics by introducing a new infectious class and established the competitive exclusion principle. Their results indicate that the basic reproduction numbers determine whether parasites can persist in the host population. When spatial structure and host movement are incorporated, Qiu et al. (2013) considered a vector–host model with multiple strains in a patchy environment and derived invasion reproduction numbers that provide threshold conditions distinguishing exclusion and coexistence under suitable assumptions. In Dénes et al. (2020), the authors studied a multistrain SIS model with superinfection and patch structure, and developed an iterative threshold procedure to establish the global asymptotic stability of the equilibria corresponding to the dominance of any subset of strains depending on parameter values. For the two-strain SIS patch model in Doumatè et al. (2024), reproduction-type thresholds also play a crucial role, while some conclusions are established under additional structural settings on dispersal/heterogeneity (and dispersal may affect the outcome beyond these settings).

Unlike these existing works, we incorporate travel loss and allow migration to be class-dependent. We investigate how the interplay among migration asymmetry, travel loss, and epidemiological traits shapes competitive exclusion and coexistence. In particular, we focus on the role of host migration and derive threshold-type conditions that characterize the circumstances under which one strain competitively excludes the other.

As is standard, the system dynamics are closely related to the basic reproduction number for each strain, denoted by $\mathcal {R}_0^k$ ($k=I,J$). As expected, if $\mathcal {R}_0^k<1$ for $k=I,J$, then both strains will eventually die out; however, if $\mathcal {R}_0^k>1$ for some $k=I,J$, the dynamics becomes much more intricate. As we will see, the strain with a larger basic reproduction number may not necessarily be dominant. This phenomenon is also observed in SIS reaction-diffusion models discussed in Ducasse and Nordmann (2023); Lou and Salako (2023).

Under the condition that $$\mathcal {R}_0^k>1$$ for some $$k=I,J$$, along with additional appropriate conditions, we establish explicit bounds for the infected migration rates (see $$m_*$$ and $$m_{**}$$ in (2.8)) that guarantee the competitive exclusion of a specific strain (Theorem 1 and Corollary 2.1). Numerical simulations complement these mathematical results. Although these bounds provide sufficient conditions for exclusion, the numerical results further illustrate how sufficiently varying the infected migration rates can ultimately change the competitive outcome. In particular, travel loss reduces the realized migrant flux and thus modifies the invasion and persistence conditions. This provides insights into how travel loss dictates the competition outcomes of pathogen strains. In addition, we also find certain conditions under which the strain whose infected class does not migrate gains a competitive dominance, depending on the movement of susceptibles (Theorem 2). Moreover, when infected individuals of both strains do not migrate, the competitive outcome is determined by reproduction numbers (Theorem 3), in line with classical single-patch exclusion results (Bremermann and Thieme 1989). Finally, we derive sufficient conditions for the existence of a coexistence equilibrium and establish its global stability (Theorem 4).

This article is organized as follows. Section 2 carries our main results, particularly regarding the competition outcomes and the possibility of coexistence of two strains. Numerical simulations are presented in Section 3. Finally, we give the concluding remark in Section 4. The proofs of the main results can be found in Appendices A to E.

## Main Results

In this section, we state our main results along with the related biological interpretations. This section is divided into three parts: the first part provides preliminary information, while the second and third parts outline our main findings.

### Preliminary

In this subsection, we give some basic results, such as the existence of an invariant set of (1.3), the basic reproduction numbers, the disease-free equilibrium, and single-strain endemic equilibria.

Hereafter, for $n\in \mathbb {N}$, we denote

$$\begin{aligned}\mathbb {R}^n_{\ge 0}=\{(x_1,\ldots ,x_n)\in \mathbb {R}^n:x_i\ge 0 \text{ for } \text{ each } i=1,\ldots ,n\}.\end{aligned}$$

For a subset *G* of $\mathbb {R}^n,$ denote $\textrm{int}(G)$ as the interior of *G*. For a square matrix *M*, we set

$$\begin{aligned} \rho (M)=&\max \{|\lambda |:\lambda \in \mathbb {C} \text{ is } \text{ an } \text{ eigenvalue } \text{ of } M\},\\ s(M)=&\max \{\textrm{Re}\lambda :\lambda \in \mathbb {C} \text{ is } \text{ an } \text{ eigenvalue } \text{ of } M\}. \end{aligned}$$

In (1.3), we adopt the order $(S_1,I_1,J_1,S_2,I_2,J_2)$ and denote this vector by *X*.

#### The disease-free equilibrium

To find the disease-free equilibrium of (1.3), we put $I_i=J_i=0$ for $i=1,2$ and reduce (1.3) into the following disease-free system

2.1 $$\begin{aligned} {\left\{ \begin{array}{ll} S_1'=\Lambda _1-d_1S_1-m_{21}^SS_1+m_{12}^S(1-\varepsilon _{12}^S)S_2\\ S_2'=\Lambda _2-d_2S_2-m_{12}^SS_2+m_{21}^S(1-\varepsilon _{21}^S)S_1. \end{array}\right. } \end{aligned}$$

By simple calculations, (2.1) has a unique positive equilibrium $(S^0_1,S^0_2)$, where

2.2 $$\begin{aligned} S^0_i=\dfrac{\Lambda _i(d_j+m_{ij}^S)+\Lambda _jm_{ij}^S(1-\varepsilon _{ij}^S)}{(d_i+m_{ji}^S)(d_j+m_{ij}^S)-m_{ij}^Sm_{ji}^S(1-\varepsilon _{ij}^S)(1-\varepsilon _{ji}^S)}>0,\ ~i,j=1,2,i\ne j. \end{aligned}$$

Since (2.1) is cooperative, it is well known that $(S^0_1,S^0_2)$ is globally asymptotically stable in int($\mathbb {R}_{\ge 0}^2$) (e.g., see (Hirsch 1984; Smith and Waltman 1995)). By the existence and uniqueness of $(S^0_1,S^0_2)$, there is a unique disease-free equilibrium of (1.3), denoted by

$$\begin{aligned}E_0=(S^0_1,0,0,S^0_2,0,0),\end{aligned}$$

where $S_i^0$ ($i=1,2$) is given in (2.2).

#### Well-posedness

By the fundamental theory of ODEs, we see that (1.3)–(1.4) admits a unique solution *X*(*t*) for all $t\ge 0$. The following result gives the boundedness of solutions and an invariant set.

##### Proposition 2.1

(Boundedness and Invariance) The solution of (1.3)–(1.4) is positive and bounded over $[0,\infty )$. Moreover, a compact positively invariant region is given by

$$\begin{aligned}\Gamma =\Big \{X\in \mathbb {R}^6_{\ge 0}:S_1+S_2+I_1+I_2+J_1+J_2\le \frac{\Lambda _1+\Lambda _2}{d},\ S_1\le S_1^0,\ S_2\le S_2^0\Big \},\end{aligned}$$

where $$d=\min \{d_1,d_2\}$$.

#### The basic reproduction numbers

In this subsection, we define the strain-*k* basic reproduction numbers for $k=I,J$, respectively.

To apply the next-generation matrix method (Driessche and Watmough 2002), we set

2.3 $$\begin{aligned} F^{k}&=\textrm{diag}(\beta _1^{k}S_1^0,\beta _2^{k}S_2^0), \nonumber \\&\quad V^{k}=\begin{bmatrix} d_1+\alpha _1^{k}+\gamma _1^{k}+m_{21}^{k}& -m_{12}^{k}(1-\varepsilon _{12})\\ -m_{21}^{k}(1-\varepsilon _{21})& d_2+\alpha _2^{k}+\gamma _2^{k}+m_{12}^{k}\\ \end{bmatrix},\quad k=I,J, \end{aligned}$$

where $S_i^0$ ($i=1,2$) is defined in (2.2). The strain-*k* reproduction number is defined by

2.4 $$\begin{aligned} \mathcal {R}_0^{k}=\rho (F^{k}(V^{k})^{-1}),\quad k=I,J. \end{aligned}$$

By some tedious calculations, we obtain

$$\begin{aligned} \mathcal {R}_0^{k}=\frac{B_{k}+\sqrt{B_{k}^2-{4\beta _1^{k}\beta _2^{k}S_1^0S_2^0A_{k}}}}{2A_{k}},\quad k=I,J, \end{aligned}$$

where

$$\begin{aligned} A_{k}&=(d_1+\alpha _1^{k}+\gamma _1^{k}+m_{21}^{k})(d_2+\alpha _2^{k}+\gamma _2^{k}+m_{12}^{k})-m_{21}^{k}m_{12}^{k}(1-\varepsilon _{21})(1-\varepsilon _{12}),\\ B_{k}&=\beta _1^{k}S_1^0(d_2+\alpha _2^{k}+\gamma _2^{k}+m_{12}^{k})+\beta _2^{k}S_2^0(d_1+\alpha _1^{k}+\gamma _1^{k}+m_{21}^{k}).\\ \end{aligned}$$

Then the basic reproduction number for (1.3) is defined by

$$\begin{aligned}\mathcal {R}_0=\max \{\mathcal {R}_0^{I},~\mathcal {R}_0^{J}\}.\end{aligned}$$

##### Remark 2.1

If $m_{12}^{k}=m_{21}^{k}=0$ for some $k=I,J$; namely, the strain-*k* infectious individuals cannot migrate, we define the strain-*k* reproduction number in the *i*-th patch as

2.5 $$\begin{aligned} \mathcal {R}_{0,i}^{k}=\dfrac{\beta _i^{k}S_i^0}{d_i+\alpha _i^{k}+\gamma _i^{k}},\quad i=1,2. \end{aligned}$$

Then, it is obvious that $\mathcal {R}_0^{k}$ defined by (2.4) is equivalent to

$$\begin{aligned} \mathcal {R}_0^{k}=\max \{\mathcal {R}_{0,i}^{k}:i=1,2\}, {\quad \text {if } m_{12}^{k}=m_{21}^{k}=0.} \end{aligned}$$

#### The stability of the disease-free equilibrium $E_0$

Now, we discuss the stability of $E_0$ in terms of $\mathcal {R}_0^{k}$. The local stability of $E_0$ follows immediately from (Driessche and Watmough 2002, Theorem 2), which is given as follows.

##### Proposition 2.2

((Driessche and Watmough 2002, Theorem 2)) The disease-free equilibrium $E_0$ is locally asymptotically stable if $\mathcal {R}_0<1$ and unstable if $\mathcal {R}_0>1$.

The following result establishes the global stability of the disease-free equilibrium.

##### Proposition 2.3

If $\mathcal {R}_0^I<1$ (resp. $\mathcal {R}_0^J<1$), then

$$\begin{aligned}\lim \limits _{t\rightarrow \infty }I_i(t)=0~\text{(resp. } \lim \limits _{t\rightarrow \infty }J_i(t)=0) \text{ for } \text{ each } \text{ patch } i=1,2,\end{aligned}$$

among all initial conditions $S_i(0)>0,I_i(0)\ge 0$ and $J_i(0)\ge 0$ for $i=1,2$. Moreover, if $\mathcal {R}_0<1$, then $E_0$ is globally asymptotically stable among all initial conditions $S_i(0)>0,I_i(0)\ge 0$ and $J_i(0)\ge 0$ for each patch $i=1,2$.

#### The existence of single-strain endemic equilibria

To ensure a strongly connected spatial structure and avoid technical issues associated with reducible migration matrices, we impose the following structural assumption throughout the remainder of this paper: for each class $\ell \in \{S,I,J\}$, we assume either

(i) (two-way migration) $m_{12}^\ell >0$ and $m_{21}^\ell >0$, or
(ii) (no migration) $m_{12}^\ell =m_{21}^\ell =0$.

The one-way migration case (e.g., $m_{12}^\ell >0$ and $m_{21}^\ell =0$) leads to a coupled but reducible dispersal structure, which complicates the equilibrium classification; see (Yan and Zou 2020).

A *strict strain-I endemic equilibrium* is an equilibrium of the form

$$\begin{aligned} E_{1}=(S_{1*},I_{1*},0,\; S_{2*},I_{2*},0)\quad \text{(persistence } \text{ of } \text{ strain } I \text{ in } \text{ both } \text{ patches) } \end{aligned}$$

where $S_{i*}>0$ and $I_{i*}>0$ for $i=1,2$. That is, strain *I* persists in both patches at $E_{1}$. A strict strain-*J* endemic equilibrium $E_2$ (persistence of strain *J* in both patches) can be defined similarly.

Setting $J_i\equiv 0,$ (1.3) is reduced to

2.6 $$\begin{aligned} {\left\{ \begin{array}{ll} S_1'=\Lambda _1-\beta ^I_1S_1I_1-d_1S_1-m_{21}^SS_1+m_{12}^S(1-\varepsilon _{12}^S)S_2,\quad t\ge 0,\\ I_1'=\beta ^I_1S_1I_1-(d_1+\alpha _1^I+\gamma _1^I)I_1-m_{21}^II_1+m_{12}^I(1-\varepsilon _{12})I_2,\quad t\ge 0,\\ S_2'=\Lambda _2-\beta ^I_2S_2I_2-d_2S_2-m_{12}^SS_2+m_{21}^S(1-\varepsilon _{21}^S)S_1,\quad t\ge 0,\\ I_2'=\beta ^I_2S_2I_2-(d_2+\alpha _2^I+\gamma _2^I)I_2-m_{12}^II_2+m_{21}^I(1-\varepsilon _{21})I_1,\quad t\ge 0.\\ \end{array}\right. } \end{aligned}$$

In order to find $E_{1}$, we consider the following two cases of $m_{ij}^{I}:$ (1) $m_{21}^{I}>0$ and $m_{12}^{I}>0;$ (2) $m_{21}^{I}=m_{12}^{I}=0$.

**Case (1): Two-way migration.** In this case, the migration matrix $$\begin{bmatrix} m_{ij}^{I} \end{bmatrix}$$ (setting $m_{ii}^{I}=0$) is irreducible. This allows us to apply a similar argument in (Li and Shuai 2009, Theorem 2.3 and Proposition 3.2) to conclude that if $\mathcal {R}_0^{I}>1$, then the strain-*I* infectious class is uniformly persistent (in the sense that there is an $\varepsilon >0$ such that $\liminf \limits _{t\rightarrow \infty }\min \limits _{i=1,2}I_i(t)\ge \varepsilon$ provided that $I_i(0)>0$ for each patch $i=1,2$). Moreover, from Li and Shuai (2009) we see that the one-strain model (2.6) admits a positive equilibrium $(S_{1*},I_{1*},S_{2*},I_{2*})$ and no boundary equilibrium other than the disease-free equilibrium (that is, the disease either dies out in both patches or prevails in both patches). This verifies the existence of a strict strain-*I* endemic equilibrium $E_{1}$ of (1.3).

**Case (2): No migration.** When $m_{21}^I=m_{12}^I=0$, a direct algebraic computation from (2.6) yields

2.7 $$\begin{aligned} &  S_{i*}=\frac{d_i+\alpha _i^I+\gamma _i^I}{\beta _i^I}>0,\nonumber \\ &  \qquad I_{i*}=\frac{\Lambda _i-(d_i+m_{ji}^S)S_{i*}+m_{ij}^S(1-\varepsilon _{ij}^S)S_{j*}}{d_i+\alpha _i^I+\gamma _i^I}, \quad i=1,2,\ j\ne i. \end{aligned}$$

In this isolated-infection scenario, a strict strain-*I* endemic equilibrium $E_1$ exists if and only if $I_{1*}>0$ and $I_{2*}>0$. Then we can derive that $\mathcal {R}_{0,1}^I>1$ and $\mathcal {R}_{0,2}^I>1$ is a necessary condition for the existence of $E_1$.

##### Proposition 2.4

Assume that $m_{21}^I=m_{12}^I=0$. If a strict strain-*I* endemic equilibrium $E_1$ exists, then $\mathcal {R}_{0,1}^I>1$ and $\mathcal {R}_{0,2}^I>1$.

##### Remark 2.2

Note that in this no-migration case, boundary (non-strict) strain-*I* endemic equilibria, where the disease prevails in one patch but vanishes in the other (e.g., $I_{1*}>0$ but $I_{2*}=0$), may also occur. Since our subsequent analysis focuses on strict endemic equilibria in both patches, we do not pursue these boundary equilibria here.

### Criteria for strain dominance

In this subsection, we first consider the case that $\mathcal {R}_0^{I}>1$ and $\mathcal {R}_0^{J}<1$. In this situation, strain *I* persists, as stated in Proposition 2.5.

#### Proposition 2.5

If $m_{12}^{I}>0$, $m_{21}^{I}>0$ and $\mathcal {R}_0^{J}<1<\mathcal {R}_0^{I},$ then strain *I* is uniformly persistent, i.e., there is an $\varepsilon >0$ such that $\liminf \limits _{t\rightarrow \infty }\min \limits _{i=1,2}I_i(t)\ge \varepsilon .$

However, when $\mathcal {R}_0^{k}>1$ for each strain $k=I,J$, the outcome is more intricate. Numerical simulations suggest that, even if $\mathcal {R}_0^{I}>\mathcal {R}_0^{J}>1$, strain *J* can still win the competition (see Table 2 in Section 3).

To get a better understanding and to simplify the discussion, we impose the following migration regimes:

For susceptibles we impose

**(M0)**: Either $m_{12}^S=m_{21}^S=0$ (no migration of susceptibles), or $m_{12}^S>0$ and $m_{21}^S>0$ (two-way migration of susceptibles).

For infected hosts, we consider

**(M1)**: (Two-way migration for both strains) $m_{12}^k>0$ and $m_{21}^k>0$ for each strain $k\in \{I,J\}$.
**(M2)**: (One migrates, one does not) $m_{12}^I=m_{21}^I=0$, $m_{12}^J>0$ and $m_{21}^J>0$.
**(M3)**: (No migration for both strains) $m_{12}^k=m_{21}^k=0$ for each strain $k\in \{I,J\}$.

We will apply the Lyapunov function technique to establish the global stability of the strain-*I* endemic equilibrium $E_{1}$, where

$$\begin{aligned} E_{1}:=(S_{1*},I_{1*},0,S_{2*},I_{2*},0) \quad \text{ for } \text{ some } S_{i*}>0 \text{ and } I_{i*}>0 \text{ for } i=1,2. \end{aligned}$$

To establish the global stability of $E_{1}$, we need the following assumption on $E_{1}$:

**(H1)**: For each patch $i=1,2,$
  $$\begin{aligned} (\beta ^{J}_i-\beta _i^{I})S_{i*}+\big ((\alpha ^{I}_i+\gamma ^{I}_i)-(\alpha _i^{J}+\gamma _i^{J})\big )\le 0. \end{aligned}$$

Note that $E_{1}$ may not be unique; thus, **(H1)** depends on the selected $E_{1}$.

#### Remark 2.3

*(Biological interpretation of* **(H1)***)* Note that the quantity $\beta _i^{k}S_{i*}-(d_i+\alpha _i^{k}+\gamma ^{k}_i)$ can be viewed as the local growth rate of strain-*k* infected hosts in patch *i* when the susceptible level is evaluated at $S_{i*}$. Condition **(H1)** is equivalent to

$$\begin{aligned} \beta _i^{J}S_{i*}-(d_i+\alpha _i^{J}+\gamma ^{J}_i)\le \beta _i^{I}S_{i*}-(d_i+\alpha _i^{I}+\gamma ^{I}_i),\qquad i=1,2, \end{aligned}$$

which asserts that in each patch, strain *J* has no larger local invasion advantage than strain *I* under the susceptible distribution maintained by $E_1$. As a special case, if virulence ($\alpha _i^I,\alpha ^J_i,~i=1,2$) is the only trait that differs between strains, i.e., $\beta ^{I}_i=\beta _i^{J}$, $\gamma ^{I}_i=\gamma _i^{J}$ and $\alpha _i^{I}\ne \alpha _i^{J}$ for $i=1,2$, then **(H1)** reduces to $\alpha _i^{I}<\alpha _i^{J}$ for each patch $i=1,2$, which means that strain *J* is the more virulent pathogen.

#### Remark 2.4

**(H1)** does not necessarily imply that $\mathcal {R}_0^{I}\ge \mathcal {R}_0^{J}$ in general (see Table 2 in our numerical simulations), due to the effect of migration rates. However, under **(M3)**, i.e., all infectious classes do not migrate, we can relate **(H1)** to the patch-wise reproduction numbers. Indeed, if **(M3)** holds, then $S_{i*}$ can be calculated as

$$\begin{aligned} S_{i*}=\dfrac{d_i+\alpha _i^{I}+\gamma _i^{I}}{\beta _i^{I}}=\frac{S_i^0}{\mathcal {R}_{0,i}^{I}},\quad i=1,2, \end{aligned}$$

where $\mathcal {R}_{0,i}^{I}$ is defined in (2.5). Hence, **(H1)** is reduced to

$$\begin{aligned} \beta ^{J}_iS_{i*}-(d_i+\alpha _i^{J}+\gamma _i^{J})\le \beta ^{I}_iS_{i*}-(d_i+\alpha _i^{I}+\gamma _i^{I})=0, \quad i=1,2. \end{aligned}$$

i.e., $${S_i^0/\mathcal {R}_{0,i}^{I} =} S_{i*}\le (d_i+\alpha _i^{J}+\gamma _i^{J})/\beta _i^{J}=S_i^0/\mathcal {R}_{0,i}^{J}$$, or equivalently,

$$\begin{aligned} \mathcal {R}_{0,i}^{J}\le \mathcal {R}_{0,i}^{I} \quad \text{ for } \text{ each } \text{ patch } i=1,2. \end{aligned}$$

Given the strain-*I* endemic equilibrium $E_1$, the following two quantities are crucial for determining the dominance of strain *I*:

2.8 $$\begin{aligned} m_*:=m^{I}_{12}(1-\varepsilon _{12})\dfrac{I_{2*}}{I_{1*}}\quad \text{ and } \quad m_{**}:=\dfrac{m_{12}^{I}}{1-\varepsilon _{21}}\dfrac{I_{2*}}{I_{1*}}, \end{aligned}$$

where $$I_{1*}$$ and $$I_{2*}$$ are the corresponding positive components of $$E_1$$. Crucially, since these values depend only on the parameters of strain *I* and the susceptible environment, $$m_*$$ and $$m_{**}$$ are entirely independent of the parameters of *J*.

Furthermore, note that $$m_*\le m_{**}$$, and the equality holds if and only if $$\varepsilon _{12}=\varepsilon _{21}=0$$, i.e., there is no travel loss for infected hosts.

#### Sufficient conditions for movement-driven strain dominance

Now, we are ready to state our main results, which provide sufficient conditions on the migration strategies of hosts to guarantee strain-*I* dominance. Specifically, Theorem 1 establishes the global asymptotic stability of $E_{1}$:

##### Theorem 1

(Movement-driven dominance of strain *I*) Assume **(M0)** and **(M1)**. Suppose that $\mathcal {R}_0^{I}>1$, and that the migration strategies of the two strains are distinct, i.e.,

$$\begin{aligned} (m^{I}_{12}, m^{I}_{21})\ne (m^{J}_{12}, m^{J}_{21}) \end{aligned}$$

Furthermore, assume that $E_{1}$ satisfies **(H1)** and that there is a constant $\lambda \ge 0$ such that

2.9 $$\begin{aligned} m_{ij}^S(1-\varepsilon _{ij}^S)S_{j*}=\lambda m_{ij}^{I}(1-\varepsilon _{ij})I_{j*}\quad \text{ for }\quad i,j=1,2,\ i\ne j. \end{aligned}$$

Then $E_{1}$ is globally asymptotically stable in $\textrm{int}(\Gamma )$, provided that one of the following holds:

1. $$m_{21}^{J}\le m^{I}_{21}\le m_*$$ and $$m_{12}^{I}\le m^{J}_{12};$$
2. $$m_{*}\le m^{I}_{21}\le m_{**}$$, $$m_{12}^{I}\le m^{J}_{12}$$ and $$m^{I}_{21}\le m_{21}^{J};$$
3. $$m_{**}\le m^{I}_{21}\le m_{21}^{J}$$ and $$m^{J}_{12}\le m_{12}^{I}$$,

where $$m_*$$ and $$m_{**}$$ are defined in (2.8).

##### Remark 2.5

Condition (2.9) imposes a proportionality between the successful cross-patch fluxes of susceptibles and strain-*I* infected hosts at $E_1$, which is a technical assumption serving as a sufficient condition used in the construction of a Lyapunov function; as such, it is not obviously a sharp condition nor is it easily verified a priori. However, our numerical results suggest that the conclusion of Theorem 1 nevertheless holds more broadly. In particular, (2.9) naturally holds when $m_{ij}^S=0$ for $i\ne j$ (i.e., when susceptible hosts do not migrate) by simply taking $\lambda =0$.

##### Remark 2.6

Theorem 1 provides migration regimes under which infected-host movement alone determines the competitive outcome. Condition **(H1)** means that strain *J* has no patch-wise invasion advantage (Remark 2.3). While, in principle, a strain may compensate for a local disadvantage by redistributing infectives through movement, the proportionality relation (2.9) and the movement comparisons in (1)–(3) prevent strain *J* from creating a movement advantage to overcome **(H1)**. Hence, strain *J* is competitively excluded. In the next corollary, we focus on a simplified setting to emphasize the emergence of a key migration bound and its biological interpretation.

The conditions (1)–(3) in Theorem 1 are involved. To provide clearer insights, we present the following corollary. Threshold-type movement effects have also been reported in food-chain models (e.g., DeAngelis et al. (2011); Lou and Wu (2011)).

##### Corollary 2.1

Let the assumptions in Theorem 1 hold. Suppose further that $m_{12}^{I}=m_{12}^{J}$. Then $E_{1}$ is globally asymptotically stable in $\textrm{int}(\Gamma )$, provided that

2.10 $$\begin{aligned} \text{ either } m_{21}^{J}<m^{I}_{21}\le m_* \text{ or } m_{*}\le m^{I}_{21}<m_{21}^{J}. \end{aligned}$$

**Fig. 1:**
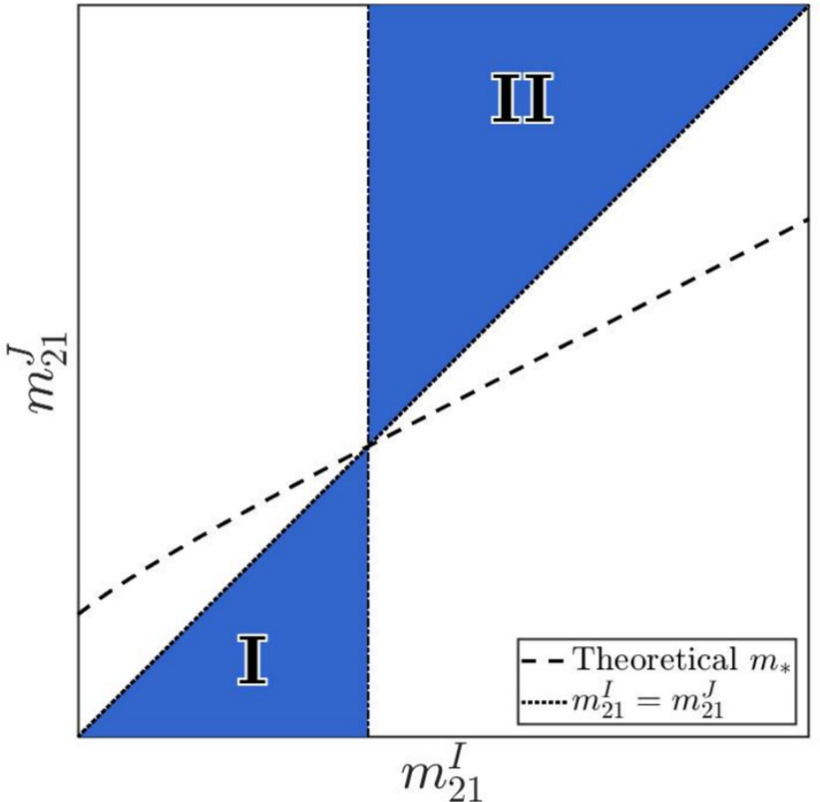
Schematic representation of the theoretical regions for strain-*I* dominance. The dashed line represents the critical migration bound $m_*=m_*(m^I_{21})$. According to Corollary 2.1, the blue shaded areas represent the regions in the parameter space where sufficient conditions are met for strain *I* to successfully exclude strain *J*. These areas are divided into Region I ($m_{21}^J < m_{21}^I \le m_*$) and Region II ($m_* \le m_{21}^I < m_{21}^J$).

Figure 1 provides a schematic representation of the regions where the competitive dominance of strain *I* is theoretically guaranteed, provided that the assumptions of Theorem 1 hold.

##### Remark 2.7

*(Biological interpretation of Corollary* 2.1*)* The assumption $m_{12}^{I}=m_{12}^J$ means that the migration rates of infected hosts from patch 2 to patch 1 are identical for both strains, which could result from factors such as water flow, prevailing wind, or policy restrictions. Under this constraint, the competitive outcome is determined by the remaining directional difference, namely, the migration rates from patch 1 to patch 2. Under **(H1)**, strain *J* is locally disadvantaged in both patches in the endemic environment created by strain *I*, and thus must rely on movement to compensate for this disadvantage. Corollary 2.1 shows that, when the movement from patch 2 to patch 1 is constrained to be identical for both strains ($m_{12}^I=m_{12}^J$), strain *J* fails to overcome this disadvantage whenever its movement rate $m_{21}^J$ from patch 1 to patch 2 lies farther from $m_*$ than $m_{21}^I$ (on the same side of $m_*$). Such a movement strategy does not allow strain *J* to create a sufficient effective directional migration advantage (after travel loss) to offset **(H1)**. Consequently, strain *J* is excluded.

#### Non-migration-driven strain dominance

In the following Theorem 2, we provide under which the non-migration strategy $(m_{12}^{I},m_{21}^{I})=(0,0)$ for strain-*I* infected hosts drives strain *J* to extinction.

##### Theorem 2

(Non-migration-driven dominance of strain *I*) Assume **(M0)**, **(M2)** and $\varepsilon _{ij}^S,\varepsilon _{ij}\in (0,1)$. Suppose that $\mathcal {R}_{0,i}^{I}>1$ for $i=1,2$, and $E_1$ satisfies **(H1)**. Then $E_{1}$ is globally asymptotically stable in $\textrm{int}(\Gamma )$, provided that

2.11 $$\begin{aligned} m_{12}^S(1-\varepsilon _{12}^S)S_{2*}(1-\varepsilon _{21})\le m_{21}^S(1-\varepsilon _{21}^S)S_{1*}\le \dfrac{m_{12}^S(1-\varepsilon _{12}^S)S_{2*}}{1-\varepsilon _{12}}, \end{aligned}$$

where $S_{i*}$, $i=1,2$, has an explicit form as evaluated in (2.7).

Note that under $m_{12}^I=m_{21}^I=0$, the condition $\mathcal {R}_{0,i}^{I}>1$ for $i=1,2$ is necessary for the existence of strict strain-*I* endemic equilibrium $E_1$ (Proposition 2.4). In addition, the global stability of $E_1$ in Theorem 2 may fail if (2.11) does not hold (see Table 3 in Section 3).

##### Remark 2.8

*(Biological interpretation of Theorem* 2*)* Note that (2.11) holds if either $m_{12}^S=m_{21}^S=0$ or the travel losses for infected populations are sufficiently large. Assume that $m_{12}^S>0$ and $m_{21}^S>0$. Then we can define $F_{12}^S:=m_{12}^S(1-\varepsilon _{12}^S)S_{2*}$ and $F_{21}^S:=m_{21}^S(1-\varepsilon _{21}^S)S_{1*}$ as the successful cross-patch fluxes of susceptibles at $E_1$. Then (2.11) is equivalent to

$$\begin{aligned} 1-\varepsilon _{21}\le \frac{F_{21}^S}{F_{12}^S}\le \frac{1}{1-\varepsilon _{12}}. \end{aligned}$$

Biologically, the ratio ${F_{21}^S}/{F_{12}^S}$ measures the directional bias of susceptible mixing at $E_1$. Since strain *I* does not migrate to compensate for local demographic changes, an excessive net loss of susceptible resources from one patch to another could destabilize the endemic state $E_1$. Therefore, condition (2.11) ensures that the cross-patch resource exchange remains sufficiently balanced. Indeed, our numerical simulations (section 3.3) reveal that if this specific bound is violated, the system may converge to a boundary equilibrium where strain *I* persists in one patch but vanishes in the other.

Finally, we consider the case $m_{12}^{k}=m_{21}^{k}=0$ for each strain $k\in \{I,J\}$ i.e., no infected hosts migrate.

##### Theorem 3

(Patch-wise dominance under non-migration) Assume **(M0)** and **(M3)**. Suppose that for each patch $i=1,2$,

$$\begin{aligned} \mathcal {R}_{0,i}^{I}>\max \{\mathcal {R}_{0,i}^{J},\,1\}, \end{aligned}$$

where $\mathcal {R}_{0,i}^{k}$ is the strain-*k* reproduction number in patch *i* with $k\in \{I,J\}$, defined in (2.5). Then $E_{1}$ is globally asymptotically stable in $\textrm{int}(\Gamma )$.

##### Remark 2.9

*(Biological interpretation of Theorem* 3*)* Theorem 3 corresponds to the situation where infected individuals do not travel (e.g., due to isolation or travel restrictions), while susceptible individuals may still move between patches. This conclusion is consistent with the competitive exclusion principle of Bremermann and Thieme (1989), and may be viewed as its spatially structured analogue under the restriction that only susceptibles migrate. However, it is crucial to note that the movement of susceptible hosts redistributes the demographic resources (i.e., the susceptible population size in each patch), which may change the local reproduction numbers $\mathcal {R}_{0,i}^k$.

Parallel results hold for the strict strain-*J* endemic equilibrium

$$\begin{aligned}E_{2}:=(S_{1*}^J,0,J_{1*},S_{2*}^{J},0,J_{2*}),\end{aligned}$$

where $S_{i*}^J>0$ for each patch $i=1,2.$

### Coexistence Endemic Equilibrium

In this subsection, we will show that (1.3) may not always follow the competitive exclusion principle, which means that coexistence of two strains is possible. To investigate coexistence state, we assume that

**(H2)**: $$\min \{\mathcal {R}_0^{I},\mathcal {R}_0^{J}\}>1$$,

so that both types of strict single-strain endemic equilibria $E_{1}$ and $E_{2}$ exist.

Let

$$\begin{aligned} \overline{F^I}(E_2)=\textrm{diag}(\beta ^I_1S^J_{1*},\beta ^I_2S^J_{2*}),\quad \overline{F^J}(E_1)=\textrm{diag}(\beta ^J_1S_{1*},\beta ^J_2S_{2*}). \end{aligned}$$

Define the strain-*I* and strain-*J* invasion numbers (Porco and Blower 1998; Iannelli et al. 2005) as

$$\begin{aligned} \overline{\mathcal {R}_0^{I}}=\overline{\mathcal {R}_0^{I}}(E_{2}):=\rho (\overline{F^{I}}(E_2)(V^{I})^{-1}),\quad \overline{\mathcal {R}_0^{J}}=\overline{\mathcal {R}_0^{J}}(E_{1}):=\rho (\overline{F^{J}}(E_1)(V^{J})^{-1}), \end{aligned}$$

respectively, where $V^I$ and $V^J$ are defined in (2.3). Then $\overline{\mathcal {R}_0^{k}}\le \mathcal {R}_0^{k}$ for each strain $k=I,J,$ since $S_{i*}\le S^0_i$ and $S_{i*}^J\le S^0_i$ for each patch $i=1,2$.

The following result provides a sufficient condition for coexistence to exist.

#### Proposition 2.6

Assume that **(M1)** and **(H2)** hold. Furthermore, assume that the following hold:

- $\min \left\{ \overline{\mathcal {R}_0^{I}}(E_{2}),\overline{\mathcal {R}_0^{J}}(E_{1})\right\} >1$ for each pair of strict endemic equilibria $E_{1}$ and $E_{2}$;
- whenever $I_1(0)>0,I_2(0)>0$ and $J_1(0)=J_2(0)=0$, we have $X(t)\rightarrow E_{1}$ as $t\rightarrow \infty$ for some strict strain-*I* endemic equilibrium $E_{1}.$
- whenever $J_1(0)>0,J_2(0)>0$ and $I_1(0)=I_2(0)=0$, we have $X(t)\rightarrow E_{2}$ as $t\rightarrow \infty$ for some strict strain-*J* endemic equilibrium $E_{2}.$

Then (1.3) has a coexistence endemic equilibrium

$$\begin{aligned} E_{3}=(S_{1\Diamond },I_{1\Diamond },J_{1\Diamond },S_{2\Diamond },I_{2\Diamond },J_{2\Diamond })\quad \text{(coexistence } \text{ of } \text{ both } \text{ strains } \text{ in } \text{ both } \text{ patches) }, \end{aligned}$$

with each component positive.

#### Remark 2.10

Conditions (b) and (c) in Proposition 2.6 are related to the global dynamics of the single-strain subsystems. Therefore, (b) (resp. (c)) holds whenever the corresponding single-strain subsystem admits a globally asymptotically stable strict endemic equilibrium. Sufficient conditions for (b) can be obtained from (Li and Shuai 2009, Theorem 4.1). In particular, (b) holds if either

(i) $m_{12}^S=m_{21}^S=0$ (no migration of susceptibles), or
(ii) the flux-balance relation holds at $E_1$:
  $$\begin{aligned} \frac{m_{12}^S(1-\varepsilon _{12}^S)}{m_{21}^S(1-\varepsilon _{21}^S)}\frac{S_{2*}}{S_{1*}} = \frac{m^{I}_{12}(1-\varepsilon _{12})}{m^{I}_{21}(1-\varepsilon _{21})}\frac{I_{2*}}{I_{1*}}. \end{aligned}$$

A sufficient condition for (c) is analogous, with *I* replaced by *J* and $E_1$ replaced by $E_2$.

Finally, we give a sufficient condition for the global stability of a coexistence endemic equilibrium.

#### Theorem 4

Suppose that **(M0)** and **(M1)** hold. Further, assume that a coexistence endemic equilibrium $E_{3}$ exists and satisfies the following flux-balance relations:

$$\begin{aligned} {\left\{ \begin{array}{ll} \dfrac{m^{I}_{12}}{m^{I}_{21}}\dfrac{I_{2\Diamond }}{I_{1\Diamond }}=\dfrac{m_{12}^{J}}{m_{21}^{J}}\dfrac{J_{2\Diamond }}{J_{1\Diamond }},& \quad \text { if }\ m_{12}^S=m_{21}^S=0,\\ \dfrac{m_{12}^S(1-\varepsilon _{12}^S)}{m_{21}^S(1-\varepsilon _{21}^S)}\dfrac{S_{2\Diamond }}{S_{1\Diamond }}=\dfrac{m^{I}_{12}(1-\varepsilon _{12})}{m^{I}_{21}(1-\varepsilon _{21})}\dfrac{I_{2\Diamond }}{I_{1\Diamond }}=\dfrac{m_{12}^{J}(1-\varepsilon _{12})}{m_{21}^{J}(1-\varepsilon _{21})}\dfrac{J_{2\Diamond }}{J_{1\Diamond }},& \quad \text { if }\ m_{21}^S>0,\ m_{12}^S>0. \end{array}\right. } \end{aligned}$$

Then $E_{3}$ is globally asymptotically stable in $\textrm{int}(\Gamma )$. In particular, $E_{3}$ is unique.

## Numerical simulations

In this section, we present some numerical examples to illustrate our theoretical results. Throughout this section, we always set the initial conditions as

3.1 $$\begin{aligned} S_i(0)=100,\quad I_i(0)=J_i(0)=5, \quad R_i(0)=0,\quad i=1,2. \end{aligned}$$

Furthermore, unless otherwise specified, we fix parameters as listed in Table 2, and the travel losses of susceptible classes are chosen as

3.2 $$\begin{aligned} \varepsilon _{12}^S=\varepsilon _{21}^S=5\times 10^{-3}. \end{aligned}$$

### Interplay between migration and travel loss on competitive outcomes

In this subsection, we perform numerical simulations to confirm the theoretical results in Corollary 2.1, and investigate the interplay between migration and travel loss on competitive outcomes. By examining the $(m_{21}^I, m_{21}^J)$ parameter plane, we numerically present a diverse range of competitive outcomes. In addition, our results illustrate that a strain with a higher reproduction number does not necessarily displace its competitor. This reveals the complex influence of migration on strain competition.

We fix the migration rates of susceptibles as

3.3 $$\begin{aligned} m_{12}^S=m_{21}^S=0\quad \text{(so } \text{ that } \text{(2.9) } \text{ holds } \text{ by } \text{ taking } \lambda =0\text{). } \end{aligned}$$

Together with the settings in Table 2 and (3.2), we calculate

3.4 $$\begin{aligned} {\left\{ \begin{array}{ll} \mathcal {R}_{0,1}^I=2.6,\hspace{2.5em}\mathcal {R}_{0,2}^I=2\hspace{3.3em}& \quad (m_{12}^I=m_{21}^I=0);\\ \mathcal {R}_{0,1}^J=2.5714,\quad \mathcal {R}_{0,2}^J=1.8919& \quad (m_{12}^J=m_{21}^J=0), \end{array}\right. } \end{aligned}$$

where $\mathcal {R}_{0,i}^{k}$ is defined in (2.5).

Under (3.4) (all infectious classes do not migrate), Theorem 3 shows that strain *I* outcompetes strain *J* in both patches. However, our numerical simulations reveal that infectious migration can reverse this outcome; specifically, strain *J* may persist or even wipe out strain *I* if condition (2.10) in Corollary 2.1 is violated.

Furthermore, we fix the migration rates of infectious classes from patch 2 to patch 1 as

3.5 $$\begin{aligned} m_{12}^I=m_{12}^J=2\times 10^{-3}. \end{aligned}$$

We have numerically confirmed that the assumption **(H1)** holds for each simulation in this subsection, within the corresponding parameter ranges specified for each case.

#### Simulation outcomes for varying $(m_{21}^I,m_{21}^J)$

First, we fix the travel losses of infectious populations as

3.6 $$\begin{aligned} \varepsilon _{12}=\varepsilon _{21}=1\times 10^{-2}. \end{aligned}$$

**Fig. 2:**
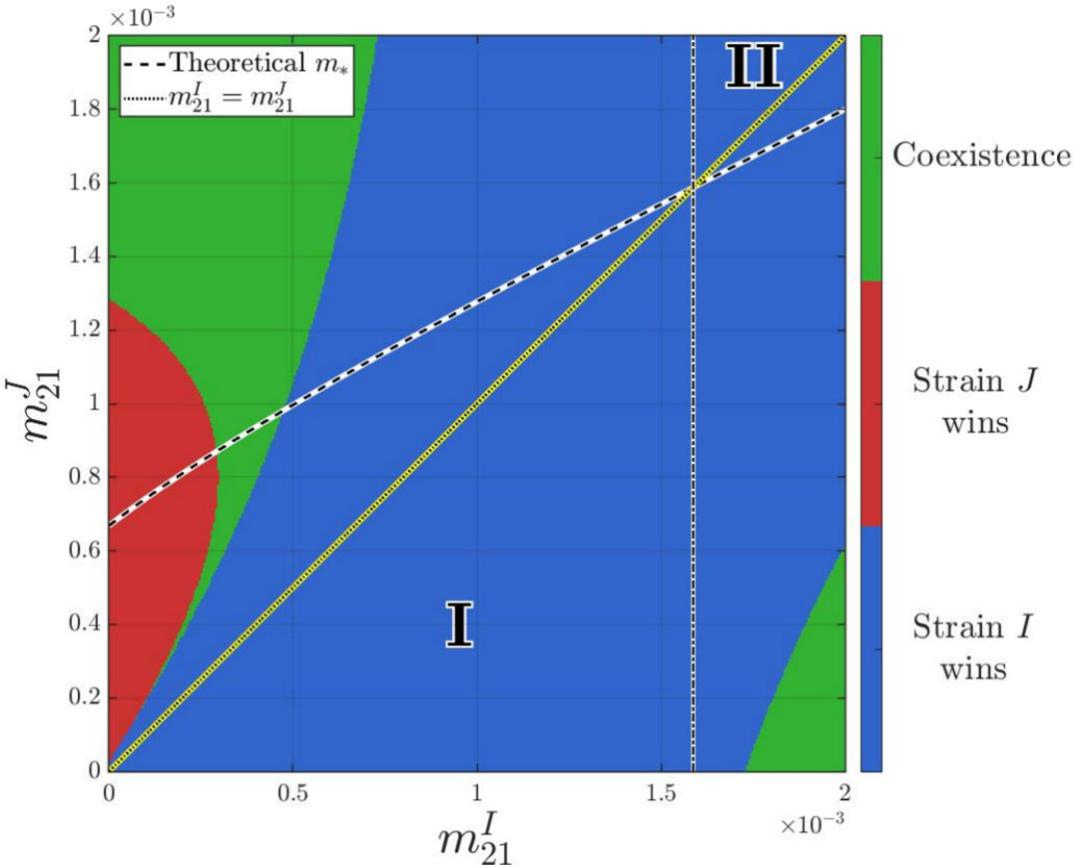
Outcome diagram for $(m_{21}^I,m_{21}^J)\in [0,~0.002]\times [0,~0.002]$ when fixing other parameters as in Table 2 and (3.2)$\sim$(3.6), in which the theoretical curve $m_*=m_*(m_{21}^I)$ (black and white dashed line) and the diagonal $m_{21}^J = m_{21}^I$ (yellow dotted line) intersect at approximately $m_{21}^I \approx 1.6 \times 10^{-3}$

We generate Figure 2 by scanning the parameter plane $(m_{21}^I,m_{21}^J)$. For each parameter pair, we numerically solve the long-time behavior of the solution to (1.3) with the initial data (3.1) and then determine the outcome by comparing the long-time sizes of the infectious populations $I_1+I_2$ and $J_1+J_2$. The color at each point $(m_{21}^I,m_{21}^J)$ records the corresponding regime (strain *I* dominance, strain *J* dominance, or coexistence). We present the resulting outcome diagram on $(m_{21}^I,m_{21}^J)\in [0,~0.002]\times [0,~0.002]$ in Figure 2, illustrating a global overview of the numerically observed dynamical outcomes.

Under the settings in Table 2 and (3.2)$\sim$(3.6), we confirm that **(H1)** holds for $(m_{21}^I,m_{21}^J)\in [0,~0.002]\times [0,~0.002]$. Consequently, Figure 2 is consistent with the conclusion of Corollary 2.1. Specifically, the theoretical curve $m_*=m_*(m_{21}^I)$ (black-and-white dashed line) and the diagonal $m_{21}^J = m_{21}^I$ (yellow dotted line) intersect at approximately $m_{21}^I \approx 1.6 \times 10^{-3}$, which divides the parameter plane into parameter regimes outlined in Corollary 2.1 (see also Figure 1):

- Region I: $m_{21}^J < m_{21}^I \le m_*$. Geometrically, this is the part of the parameter plane strictly below the diagonal and to the left of the vertical line passing through the intersection point. As shown in the figure, this region falls within the blue area, indicating that strain *I* excludes strain *J*.
- Region II: $m_* \le m_{21}^I < m_{21}^J$. This is the part above the diagonal and to the right of the vertical line passing through the intersection point. This region also falls entirely within the blue area, where strain *I* excludes strain *J*.

Outside these two regimes, Corollary 2.1 does not necessarily apply. Numerically, these parameter regions provide opportunities for strain *J* to persist, and lead to strain-*J* dominance or coexistence with strain *I*.

**Fig. 3:**
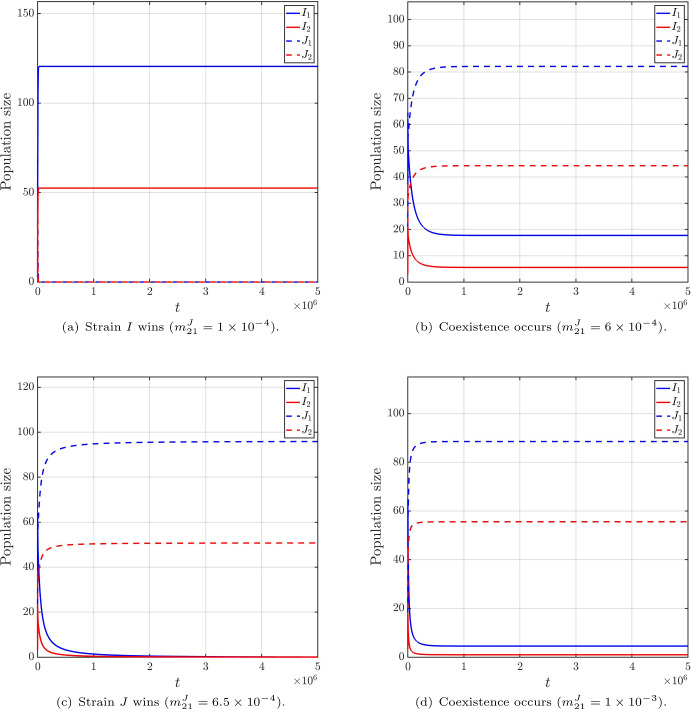
The infection sizes for four values of $m_{21}^I$, with other parameters fixed as in Table 2 and (3.2)$\sim$(3.6), and the initial condition (3.1)

Furthermore, Figure 2 reveals a non-monotonic transition of outcomes. If we fix $m_{21}^I=2.8\times 10^{-4}$, then $m_*=m_*(m_{21}^I)$ can be calculated as $m_*\approx 8.6\times 10^{-4}$. By varying four values of $m_{21}^J\in \left\{ 1\times 10^{-4},6\times 10^{-4},6.5\times 10^{-4},1\times 10^{-3}\right\}$, we can trace a horizontal cross-section of the parameter phase diagram. The numerical results are summarized in Table 2, with the corresponding population sizes of infectious individuals displayed in Figure 3. These results reveal that, as $m_{21}^J$ increases: strain *I* wins $\rightarrow$ coexistence $\rightarrow$ strain *J* wins $\rightarrow$ coexistence. Additionally, the strain with the larger reproduction number may not able to displace its competitor.

#### The impact of travel losses

**Fig. 4:**
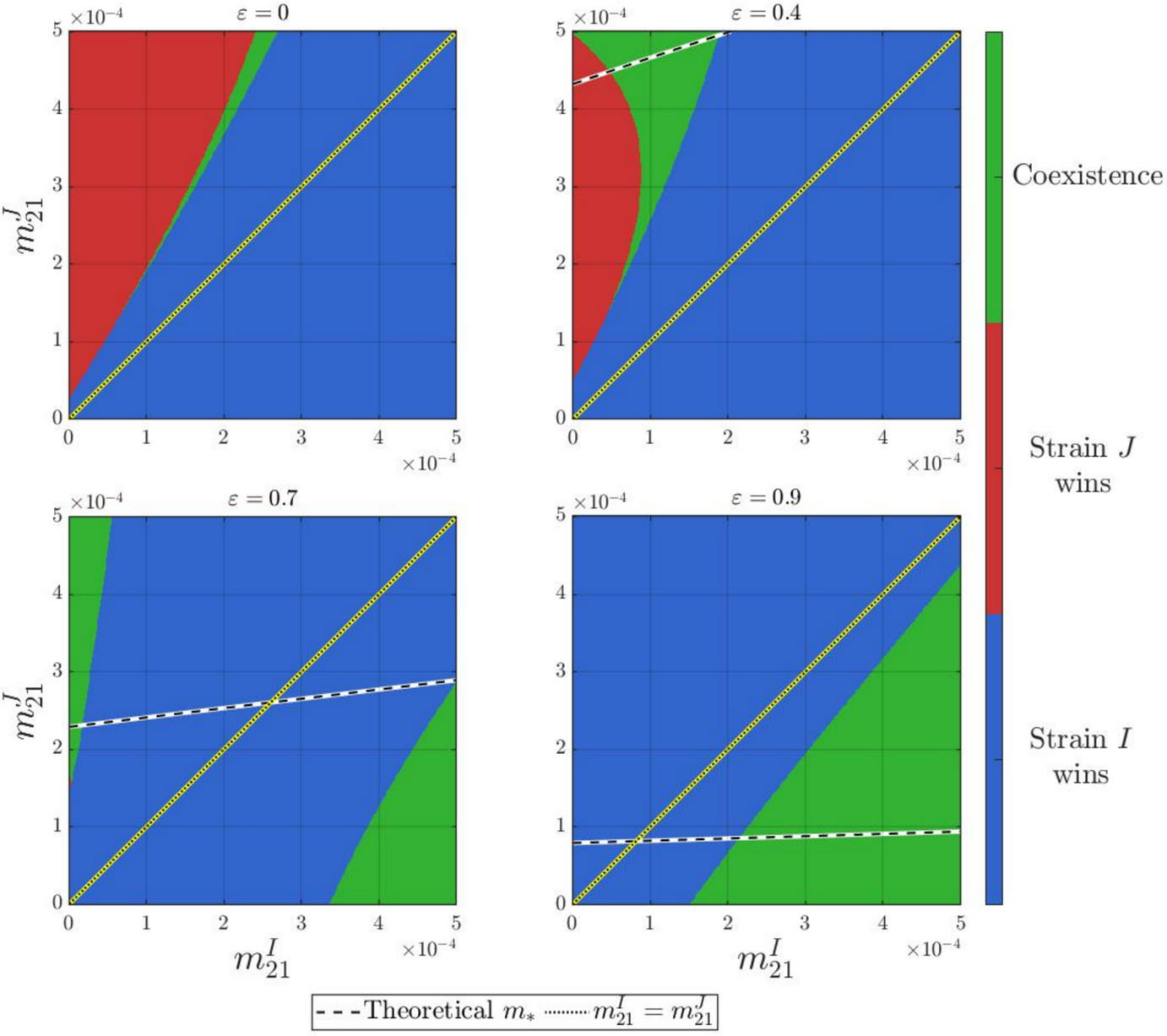
The outcome diagrams for four values of $\varepsilon =\varepsilon _{12}=\varepsilon _{21}\in \left\{ 0,0.4,0.7,0.9\right\}$, with other parameters fixed as in Table 2 and (3.2)$\sim$(3.5). Here the black and white dashed curve $m_*=m_*(m_{21}^I)$ is not shown in the diagram for $\varepsilon =0$ because it lies completely above the plotted region $(m_{21}^I,m_{21}^J)\in [0,~0.0005]\times [0,~0.0005]$

Under the settings in Table 2 and (3.2)$\sim$(3.5), we vary the travel losses

$$\begin{aligned}\varepsilon :=\varepsilon _{12}=\varepsilon _{21}\in \{0,0.4,0.7,0.9\}\end{aligned}$$

to get four outcome diagrams for $(m_{21}^I,m_{21}^J)\in [0,~0.0005]\times [0,~0.0005]$, as shown in Figure 4. Among these four outcome diagrams, we confirm that **(H1)** holds for each of these four values of $\varepsilon$ when $m_{21}^I\in [0,~0.0005].$

**Table 2:** Simulation outcomes for varying $m_{21}^J$ with $m_{21}^I=2.8\times 10^{-4}$ .

$m_{21}^J$	$1\times 10^{-4}$	$6\times 10^{-4}$	$6.5\times 10^{-4}$	$1\times 10^{-3}$
Does (2.10) hold?	Yes	No	No	No
$\mathcal {R}_0^I$	2.5517	2.5517	2.5517	2.5517
$\mathcal {R}_0^J$	2.5532	2.4708	2.4633	2.4143
Winner	Strain *I*	Coexistence	Strain *J*	Coexistence

Within the range $m_{21}^I\in [0,~0.0005],$ we observe that when the travel loss $\varepsilon$ is small ($\varepsilon =0,~\varepsilon =0.4$), strain *I* dominates provided that strain *I* hosts migrate more frequently than strain *J* (i.e., $m_{21}^J< m_{21}^I < m_*$). However, this pattern is disrupted under large travel losses ($\varepsilon =0.7,~\varepsilon =0.9$). For example, the system exhibits coexistence of two strains when $\varepsilon =0.9$ and $(m_{21}^I,m_{21}^J)=(4\times 10^{-4},~1\times 10^{-4})$, in which case the condition (2.10) in Corollary 2.1 is not satisfied. This outcome is biologically reasonable. Although assumption **(H1)** ensures that strain *I* has a strict patch-wise advantage, large travel losses heavily penalize its relatively frequent migration. This penalty effectively diminishes strain *I*’s overall competitive edge, which allows the locally weaker strain *J* to persist and coexist.

In summary, our numerical simulations in § 3.1 lead to the following conclusions:

- **Predictability of competitive exclusion:** The value $m_*$ serves as an accurate criterion for determining strain-*I* dominance, when the assumption **(H1)** holds.
- **Non-monotonic transitions of dynamical outcomes:** When migration rates shift significantly (e.g., $m_{21}^I$ is small and $m_{21}^J$ is sufficiently large), the system moves outside the sufficient condition (2.10) of Corollary 2.1 (see also Figure 1), leading to complex, non-monotonic transitions between competitive exclusion and coexistence.
- **Trade-off between migration and travel loss:** The assumption **(H1)** indicates that strain *I* has local competitive advantage over strain *J* in both patches. Thus, strain *J* can compensate for this local disadvantage only through migration. In the case of small travel losses, strain *J* is able to persist, even wipe out strain *I* when the hosts adopt favorable migration conditions. However, large travel losses penalize movement directly. As the travel losses are large, strain *J* can only persist if its hosts migrate at a low rate while strain-*I* hosts migrate more frequently than strain-*J* hosts.

### Robustness under small deviations from (2.9)

In this subsection, we examine the robustness of the conclusions of Theorem 1 (or Corollary 2.1) when the condition (2.9) is violated. We fix

3.7 $$\begin{aligned} m_{12}^S=2.5\times 10^{-3},\quad m_{21}^S=2\times 10^{-3},\quad m_{12}^I=m_{12}^J=2\times 10^{-3},\quad m_{21}^J=2\times 10^{-3} \end{aligned}$$

and

3.8 $$\begin{aligned} \varepsilon _{12}=\varepsilon _{21}=1\times 10^{-2}. \end{aligned}$$

We vary $m_{21}^I\in [0,0.002]$. For each $m_{21}^I$, we numerically compute the corresponding equilibrium $E_{1}(m_{21}^I)$ and evaluate

$$\begin{aligned} \lambda _{12}=\lambda _{12}(m_{21}^I):=\dfrac{m_{12}^S(1-\varepsilon _{12}^S)S_{2*}}{m_{12}^I(1-\varepsilon _{12})I_{2*}},\quad \lambda _{21}=\lambda _{21}(m_{21}^I):=\dfrac{m_{21}^S(1-\varepsilon _{21}^S)S_{1*}}{m_{21}^I(1-\varepsilon _{21})I_{1*}}. \end{aligned}$$

Note that assumption **(H1)** holds throughout this range of $m_{21}^I$.

**Fig. 5:**
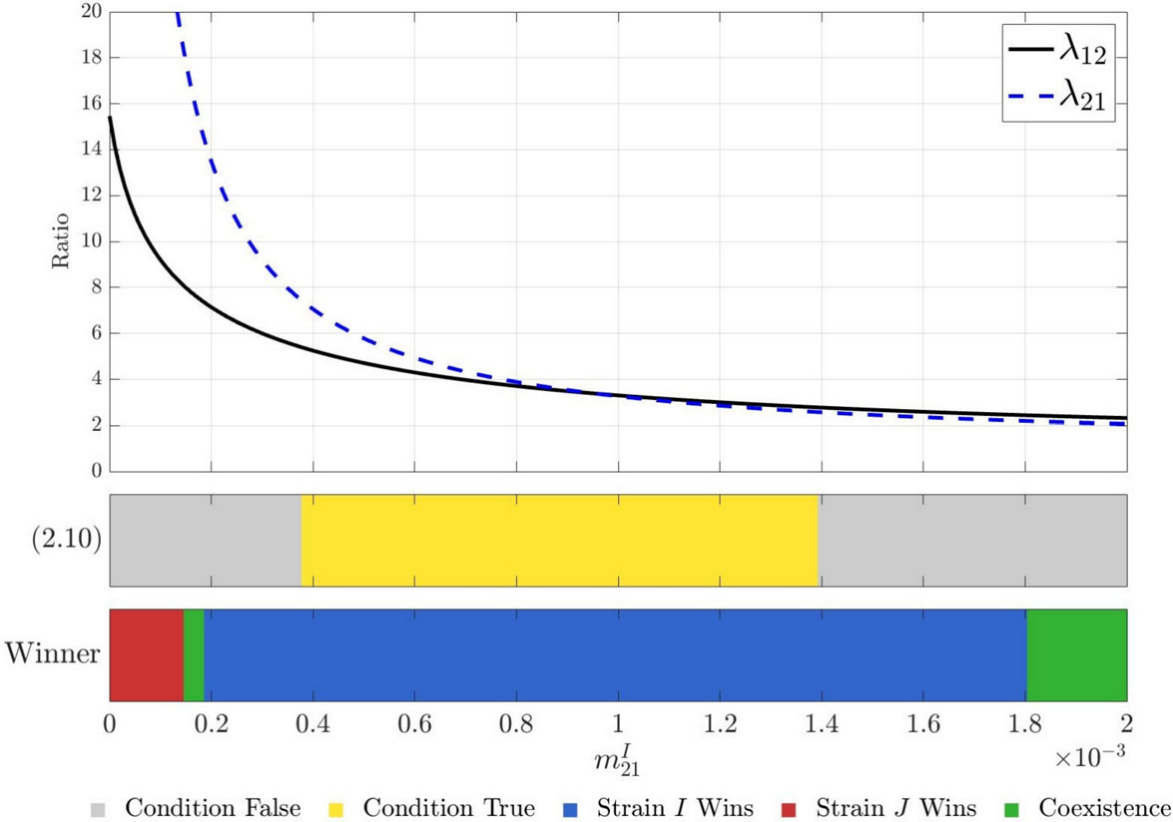
Effect of $m_{21}^I$ on competitive outcomes, with other parameters fixed as in Table 2, (3.2), (3.7) and (3.8)

In Figure 5, we plot the graphs of functions $\lambda _{12}(m_{21}^I)$ and $\lambda _{21}(m_{21}^I)$, and examine the validity of condition (2.10), in which we use yellow to denote that the conditions are satisfied, and gray to indicate that they are not. Furthermore, we present the competitive outcomes derived from numerical simulations to illustrate the transition between competitive exclusion and coexistence.

We can see from Figure 5 that

$$\begin{aligned}\lambda _{12}=\lambda _{21}\text{, } \text{ i.e., } \text{(2.9) } \text{ holds, } \text{ at } m_{21}^I\approx 9.5\times 10^{-4}.\end{aligned}$$

Despite $\lambda _{12} \ne \lambda _{21}$ for nearby values of $m_{21}^I$, the numerical results show that strain *I* still wins the competition on the interval of $m_{21}^I$ in which (2.10) remains satisfied (yellow bar). This suggests that the conclusion of Theorem 1 (or Corollary 2.1) is robust with respect to small deviations from the condition (2.9).

### Competitiveness of the non-migration strategy

In this subsection, we examine whether the non-migration strategy of strain-*I* infected hosts can allow strain *I* to outcompete strain *J* in both patches. Specifically, we fix

3.9 $$\begin{aligned} m_{12}^I=m_{21}^I=0, \end{aligned}$$

meaning that strain-*I* infectious populations do not migrate. Furthermore, we fix

3.10 $$\begin{aligned} m_{12}^S=1.5\times 10^{-3},\quad m_{12}^J=m_{21}^J=2\times 10^{-3},\quad \varepsilon _{12}=\varepsilon _{21}=1\times 10^{-2}. \end{aligned}$$

Then the left-hand sides of **(H1)** are $-7.7\times 10^{-5}$ for $i=1,$ and $-4\times 10^{-4}$ for $i=2,$ and thus **(H1)** holds.

**Fig. 6:**
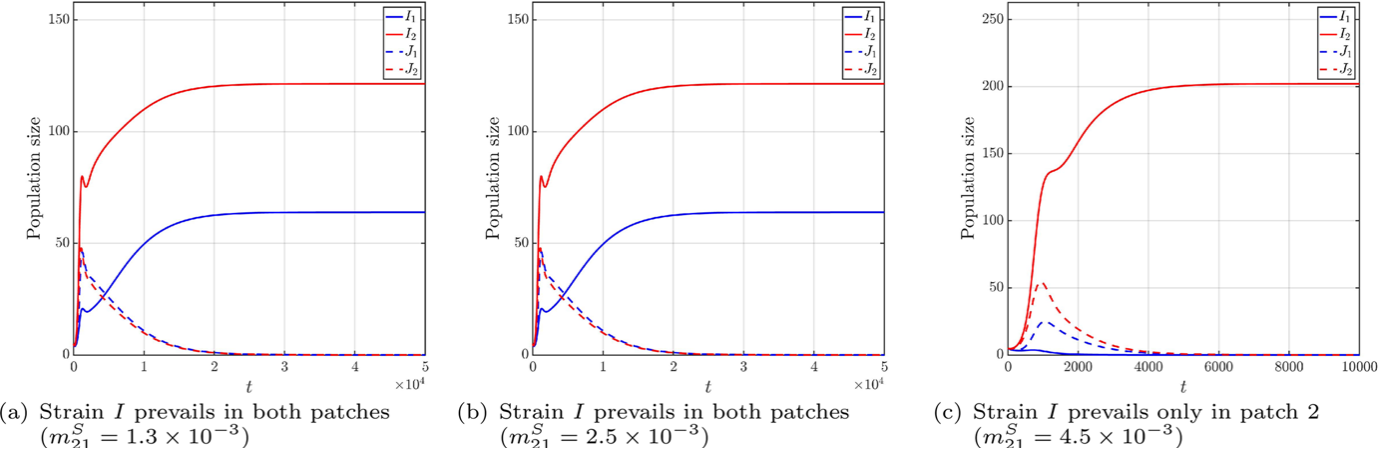
The infection sizes for three values of $m_{21}^S$, with other parameters fixed as in Table 2, (3.2), (3.9) and (3.10), and the initial condition (3.1).

We vary three values of $m_{21}^S$, and the numerical results are summarized in Table 3, with the corresponding population sizes of infectious individuals displayed in Figure 6.

**Table 3:** Outcomes of simulations for the non-migration strategy.

Does (2.11) hold?	Yes	No	No
$\mathcal {R}_{0,1}^I$	2.4119	1.8843	1.3808
$\mathcal {R}_{0,2}^I$	2.1333	2.5352	2.9186
$\mathcal {R}_{0}^J$	2.2176	2.1882	2.3725
Outcomes of strain *I*	prevails in both patches	prevails in both patches	prevails only in patch 2

In the case $m_{21}^S=1.3\times 10^{-3}$, we see that (2.11) holds, and strain *I* outcompetes strain *J* in both patches, in agreement with Theorem 2. However, once (2.11) fails, strain *I* may still outcompete strain *J* in both patches ($m_{21}^S=2.5\times 10^{-3}$), or dies out in one patch and prevails in another patch ($m_{21}^S=4.5\times 10^{-3}$). That is, the non-migration strategy may not allow strain *I* to prevail in both patches, even if the strain-*I* reproduction numbers in both patches are greater than 1. This phenomenon is biologically reasonable, as a too large migration rate $m_{21}^S$ of susceptibles from patch 1 to patch 2 violates the susceptible host balance maintained by condition (2.11). This causes patch 1 to “run out of resource,” while strain-*I* hosts cannot migrate to track the spatial redistribution of susceptible hosts. This results in a spatial mismatch, where strain *I* remains trapped in a resource-depleted environment, leading to local extinction despite its high reproductive potential.

### Migration under asymmetric patch quality

In this subsection, we investigate the competitive dynamics of system (1.3), where each strain possesses a distinct favorable patch. Moreover, **(H1)** does not hold in this setting, so Theorem 1 and Corollary 2.1 do not necessarily apply.

We fix all parameters as in Table 2, except for the transmission rates:

$$\begin{aligned} \beta _1^I=1.1\times 10^{-5},\quad \beta _2^I=2.6\times 10^{-5},\quad \beta _1^J=2.5\times 10^{-5},\quad \beta _2^J=2.1\times 10^{-5}. \end{aligned}$$

Furthermore, we fix

$$\begin{aligned} m_{12}^S=m_{21}^S=0. \end{aligned}$$

In this case,

$$\begin{aligned} \mathcal {R}_{0,1}^I=0.9167,\quad \mathcal {R}_{0,2}^I=1.4444\quad (m_{12}^I=m_{21}^I=0);\\ \mathcal {R}_{0,1}^J=1.7857,\quad \mathcal {R}_{0,2}^J=0.9459\quad (m_{12}^J=m_{21}^J=0). \end{aligned}$$

Note that this environment is characterized by opposing local suitability. Specifically, without the infective movement, strain *I* is unable to persist in patch 1 (since $\mathcal {R}_{0,1}^I < 1$) but thrives in patch 2 (since $\mathcal {R}_{0,2}^I > 1$). Conversely, strain *J* thrives in patch 1 but cannot sustain itself in patch 2 without inflow.

Now, we assume that

$$\begin{aligned} m_{12}^I=m_{12}^J=2\times 10^{-3},\quad \varepsilon _{12}=\varepsilon _{21}=1\times 10^{-2}. \end{aligned}$$

**Fig. 7:**
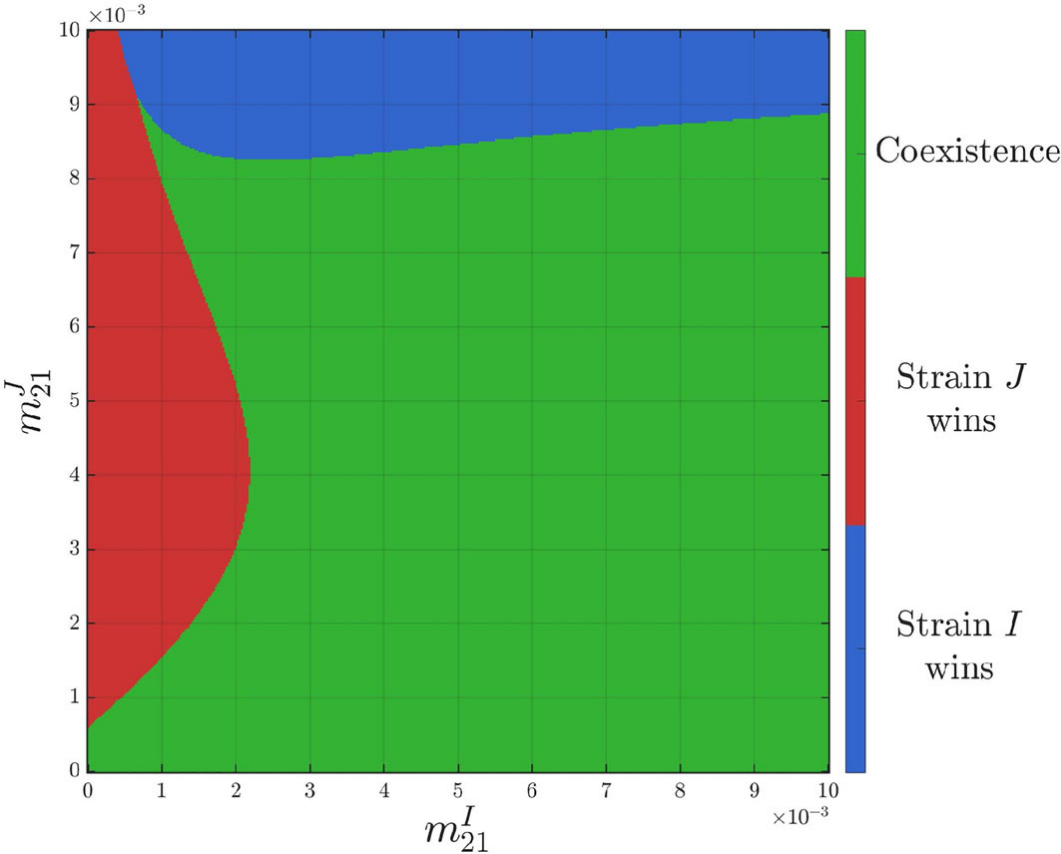
Outcome diagram for $(m_{21}^I,m_{21}^J)\in [0,~0.01]\times [0,~0.01]$ under asymmetric patch quality.

Under these settings, we draw a outcome diagram of $(m_{21}^I,m_{21}^J)$ as in Figure 7.

We summarize our findings in Figure 7 as follows.

- **Transition to coexistence driven by spatial rescue:** For moderate values of $m_{21}^J$ (approximately $1\times 10^{-3}<m_{21}^J<8\times 10^{-3}$), as $m_{21}^I$ increases, there is a transition from strain-*J* dominance to coexistence. Biologically, when $m_{21}^I$ is low, strain *I* hosts are confined in the sink habitat (patch 1) and cannot effectively relocate to its favorable patch (patch 2). Hence, strain *I* may suffer extinction. However, an increased migration rate $m_{21}^I$ may provide a spatial rescue effect such that strain *I* hosts persist in patch 2. This makes coexistence with strain *J* possible.
- **Exclusion of strain** *J* **due to source depletion:** For large $m_{21}^J$ (approximately $m_{21}^J>9\times 10^{-3}$), as $m_{21}^I$ increases, there is a transition from strain-*J* dominance to strain-*I* dominance. In this case, excessive emigration of strain *J* from its favorable patch (patch 1) poses a negative effect on its persistence, while sufficiently large $m_{21}^I$ allows strain *I* to concentrate and persist in its favorable patch (patch 2), which leads to the exclusion of strain *J*.

## Discussion

In this paper, we investigate the effect of migration rates on the competitive dynamics of two infectious strains using a two-strain SIR epidemic model with travel loss in patchy environments. The concept of travel loss has been widely studied in ecological models; see, for example, Bonte et al. (2012) for a broad review, DeAngelis et al. (2011) for an explicit travel-loss framework, Klaassen et al. (2014) for empirical evidence of mortality during migration and mathematical studies (Galanthay 2015; Lou and Wu 2011; Wu 2016).

We extended this ecological concept to a two-strain epidemiological framework. Unlike traditional ecological models, where resources are often stationary or follow growth laws, the “resources” in our model (the susceptible hosts) are themselves spatially dynamic. Their continuous cross-patch movement actively redistributes the potential for infection, which makes the role of infective migration in the competitive outcome more elusive.

Our analytical and numerical results reveal a fundamental trade-off in the dispersal of infected hosts. On the one hand, migration helps a strain spread to more places. On the other hand, travel loss penalizes movement directly. Crucially, the concurrent migration of susceptible hosts redistributes the essential resources for infection and thus fundamentally alters the quality of each patch. Consequently, the ultimate competitive outcome is governed by a complex interplay among the benefits of dispersal, the associated mortality costs, and the spatial distribution of resources.

Under assumption **(H1)**, strain *J* has no intrinsic invasion advantage over strain *I* under the susceptible population distribution maintained by $E_1$, so any advantage must come from dispersal. We rigorously established sufficient conditions that guarantee the global dominance of strain *I* (Theorem 1 and Corollary 2.1), which means in these regimes, strain *J* is unable to reverse its disadvantage. However, our numerical results suggest that strain *J* may overcome its local biological disadvantage by adjusting its migration rates, e.g., shifting them toward specific regimes such that the condition (2.9) is violated.

We also focus on the non-migration strategy for infected hosts, which provides a different trade-off. When susceptible flux remains balanced (or travel losses of infectious classes are sufficiently high), non-migration of strain-*I* infectives can secure dominance in both patches (Theorem 2). In contrast, when susceptible movement becomes strongly biased, our numerical simulation suggests that non-migration may create a spatial mismatch and strain *I* may persist in one patch and die out in another patch.

Furthermore, we examined a setting where each strain has its own locally favorable patch, so that **(H1)** no longer holds. Although the results derived under **(H1)** are not guaranteed to apply, the numerical outcome diagram reveals a reasonable biological mechanism: emigration from an unfavorable patch can prevent global extinction by allowing a strain to persist in a favorable patch and reshape dominance.

Finally, we tested various initial conditions in parameter regimes where multiple equilibria might exist, though we did not observe bistability in our current simulations (data not shown). Multistability cannot be excluded in general, and we leave it for future work. Beyond the two-patch setting, it will be important to extend the analysis to multi-patch or network-coupled environments and to state-dependent dispersal strategies. Such extensions would not only deepen our understanding of how dispersal strategies operate in more complex spatial settings, but also help connect our results to intervention design in more realistic spatial systems.

## Data Availability

Data sharing does not apply to this article because no datasets were generated or analyzed during the current study.

## Appendix A Proofs of Propositions 2.1, 2.3 and Proposition 2.4

In this section, we provide the proofs of the basic results. We first check the boundedness of solutions of (1.3).

### Proof of Proposition 2.1

We denote $\textrm{bd}(\mathbb {R}^2_+)$ as the boundary of $\mathbb {R}^2_+.$ Suppose that there is a smallest $t_1>0$ such that $(S_1(t_1),S_2(t_1))\in \textrm{bd}(\mathbb {R}^2_+),$ say $S_1(t_1)=0$ and $S_2(t_1)\ge 0$. Then $S_1'(t_1)\le 0$. However, by the equation of $S_1$, we have

$$\begin{aligned}S_1'(t_1)\ge \Lambda _1+m_{12}^S(1-\varepsilon _{12}^S)S_2(t_1)> 0\end{aligned}$$

by the choice of $t_1.$ This reaches a contradiction. Next, we solve the equation of $I_i,J_i$ as follows.

$$\begin{aligned} I_i(t)&=\exp \bigg (-\int _0^tp^I(s)ds\bigg )\bigg [\int _0^t\exp \bigg (\int _0^sp^I(\tau )d\tau \bigg )m_{ij}^{I}(1-\varepsilon _{ij})I_j(s)ds+I_i(0)\bigg ],\\ J_i(t)&=\exp \bigg (-\int _0^tp^J(s)ds\bigg ) \bigg [\int _0^t\exp \bigg (\int _0^sp^J(\tau )d\tau \bigg )m_{ij}^{J}(1-\varepsilon _{ij})J_j(s)ds+J_i(0)\bigg ],~i\ne j, \end{aligned}$$

where

$$\begin{aligned} p^k(t)={-}\beta _i^{k}S_i(t){+}(d_i+\alpha _i^{k}+\gamma ^{k}_i+m_{ji}^{k}),\quad k=I,J. \end{aligned}$$

Since $I_i(0)>0$ and $J_i(0)>0,$ we see that $I_i(t)>0$ and $J_i(t)>0$ for any $t>0$.

To derive an upper bound of the solution, we set $N(t):=S_1(t)+S_2(t)+I_1(t)+I_2(t)+J_1(t)+J_2(t)$. Then, summing up the equations in (1.3), one has

A.1 $$\begin{aligned} N'(t)\le (\Lambda _1+\Lambda _2)-dN(t), \end{aligned}$$

where $d=\min \{d_1,d_2\}$. The differential inequality gives

$$\begin{aligned} \limsup \limits _{t\rightarrow \infty }N(t)\le \frac{\Lambda _1+\Lambda _2}{d}. \end{aligned}$$

Therefore, the solution is positive and bounded for $t\ge 0$.

Finally, we construct an invariant set. Note that

$$\begin{aligned} {\left\{ \begin{array}{ll} S_1'\le \Lambda _1-d_1S_1-m_{21}^SS_1+m_{12}^S(1-\varepsilon _{12}^S)S_2\\ S_2'\le \Lambda _2-d_2S_2-m_{12}^SS_2+m_{21}^S(1-\varepsilon _{21}^S)S_1. \end{array}\right. } \end{aligned}$$

Since $m_{ij}^S$ and $1-\varepsilon _{ij}^S$ are nonnegative, we observe that the disease-free system (2.1) is of type *K* (Hsu and Chen 2022, Definition 2.6.1). By Kamke’s Theorem (Hsu and Chen 2022, Theorem 2.6.3), if $(\overline{S_1},\overline{S_2})$ is a solution of (2.1) with the same initial condition of the susceptible numbers of the original system (1.3), then $S_i(t)\le \overline{S_i}(t)$ for all $t\ge 0$ and $i=1,2.$ In particular, if $S_i(0)\le S_i^0,$ then $S_i(t)\le S_i^0$ for all $t\ge 0$, where $S_i^0$ is defined in (2.2). Together with (A.1), we see that $\Gamma$ is a compact positively invariant set. This completes the proof. $\square$

Next, we establish the global stability of the disease-free equilibrium $E_0.$

### Proof of Proposition 2.3

For convenience, we write

$$\begin{aligned} \textbf{X}^I(t)=(I_1(t),I_2(t))^T,\quad \textbf{X}^J(t)=(J_1(t),J_2(t))^T,\quad \textbf{X}(t)=( \textbf{X}^I(t), \textbf{X}^J(t))^T \end{aligned}$$

The proof is based on a comparison argument. Recall that the unique positive equilibrium $(S_1^0,S_2^0)$ of (2.1) is globally asymptotically stable in int($\mathbb {R}_{\ge 0}^2$). Simple comparison implies that for any $\varepsilon >0$, there exists $T\gg 0$ such that $S_i(t)<S_i^0+\varepsilon$ for all $t\ge T$ and $i=1,2$. Therefore, one has

A.2 $$\begin{aligned} \dot{\textbf{X}}^{k}<(F^{k}_{\varepsilon }-V^{k})\textbf{X}^{k},\quad t\ge T,\quad k=I,J, \end{aligned}$$

where $F^{k}_{\varepsilon }:=\textrm{diag}\Big (\beta _1^{k}(S_1^0+\varepsilon ),\beta _2^{k}(S_2^0+\varepsilon )\Big ),~k=I,J$. Recall the fact that $s(F^{k}-V^{k})<0$ if and only if $\mathcal {R}^{k}_0<1$ (Driessche and Watmough 2002, Theorem 2). Whenever $\mathcal {R}_0^k<1$ for some strain $k=I,J$, one can choose $\varepsilon >0$ sufficiently small such that $s(F^{k}_{\varepsilon }-V^{k})<0$. Note that for each strain $k=I,J$, the system

A.3 $$\begin{aligned} \dot{\textbf{Y}}^{k}=(F^{k}_{\varepsilon }-V^{k})\textbf{Y}^{k},\quad \textbf{Y}^{k}(T)=\textbf{X}^{k}(T), \end{aligned}$$

is of type *K* (Hsu and Chen 2022, Definition 2.6.1) and has a unique solution $\textbf{Y}^{k}(t)=e^{(F^{k}_{\varepsilon }-V^{k})(t-T)}\textbf{X}^{k}(T)$. Since $s((F^{k}_{\varepsilon }-V^{k}))<0,$ we see that $\lim \limits _{t\rightarrow \infty } \textbf{Y}^{k}(t)=\textbf{0}.$ By Kamke’s Theorem (Hsu and Chen 2022, Theorem 2.6.3), $\textbf{0}\le \textbf{X}^{k}(t)\le \textbf{Y}^{k}(t)$ for all $t\ge T.$ Thus, $\lim \limits _{t\rightarrow \infty } \textbf{X}^{k}(t)=\textbf{0}$.

Now, we assume that $\mathcal {R}_0<1$. Then $\lim \limits _{t\rightarrow \infty } \textbf{X}^{k}(t)=\textbf{0}$ for each strain $k=I,J$. Since $S_i(t)$ is bounded for $i=1,2,$ for any small $\varepsilon >0,$ we can choose $T_1\gg 0$ such that for each $i=1,2,$ and $t>T_1,$

$$\begin{aligned}\left| \beta _i^{I}S_i(t)I_i(t)+\beta _i^{J}S_i(t)J_i(t)\right| <\varepsilon .\end{aligned}$$

Then for all $t>T_1,$

A.4 $$\begin{aligned} \Lambda _i-\varepsilon -(d_i+m_{ji}^S)S_i+m_{ij}^S(1-\varepsilon _{ij}^S)S_j\le S_i'(t)\le \Lambda _i-d_iS_i-m_{ji}^SS_i+m_{ij}^S(1-\varepsilon _{ij}^S)S_j. \end{aligned}$$

Let $(\overline{S_1}(t),\overline{S_2}(t))$ be the solution of (2.1) with initial conditions $(S_1(T_1),S_2(T_1))$ at $t=T_1$; also, let $(\underline{S_1}(t),\underline{S_2}(t))$ be the solution of (2.1) with $\Lambda _i$ replaced by $\Lambda _i-\varepsilon$ and the same initial conditions at $t=T_1$. Since these two systems are of type *K*, with (A.4), we see that for all $t>T_1,$ $\underline{S_i}(t)\le S_i(t)\le \overline{S_i}(t)$.

Let $(S_{1,\varepsilon },S_{2,\varepsilon })$ be the unique positive equilibrium of the system (2.1) with $\Lambda _i$ replaced by $\Lambda _i-\varepsilon$, which is globally asymptotically stable in $\textrm{int}(\mathbb {R}^2_{\ge 0})$. Then

$$\begin{aligned}S_{i,\varepsilon }\le \liminf \limits _{t\rightarrow \infty }S_i(t)\le \limsup \limits _{t\rightarrow \infty }S_i(t)\le S_i^0.\end{aligned}$$

Since $\lim \limits _{\varepsilon \rightarrow 0^+}S_{i,\varepsilon }=S_i^0,$ we see that $\lim \limits _{t\rightarrow \infty }S_i(t)=S_i^0$. Together with Proposition 2.2, we conclude that $E_0$ is globally asymptotically stable. Thus, the proof is complete. $\square$

### Remark A.1

We can also apply the Lyapunov function technique as used in (Li and Shuai 2009, Theorem 3.1) to conclude that if $\mathcal {R}_0\le 1$, then $E_0$ is globally asymptotically stable in $\Gamma$.

### Proof of Proposition 2.4

First, from (2.5) and (2.7), we have

A.5 $$\begin{aligned} S_{i*}=\dfrac{d_i+\alpha _i^{I}+\gamma _i^{I}}{\beta _i^{I}}=\frac{S_i^0}{\mathcal {R}_{0,i}^{I}},\quad i=1,2, \end{aligned}$$

Assume that a strict strain-*I* endemic equilibrium $E_1$ exists, which implies $I_{1*}>0$ and $I_{2*}>0$. Therefore,

A.6 $$\begin{aligned} {\left\{ \begin{array}{ll} \Lambda _1 - (d_1 + m_{21}^S)S_{1*} + m_{12}^S(1-\varepsilon _{12}^S)S_{2*}> 0, \\ \Lambda _2 - (d_2 + m_{12}^S)S_{2*} + m_{21}^S(1-\varepsilon _{21}^S)S_{1*} > 0. \end{array}\right. } \end{aligned}$$

Recall the conditions at the disease-free equilibrium $(S_1^0, S_2^0)$

$$\begin{aligned} \Lambda _1 = (d_1 + m_{21}^S)S_1^0 - m_{12}^S(1-\varepsilon _{12}^S)S_2^0, \quad \Lambda _2 = (d_2 + m_{12}^S)S_2^0 - m_{21}^S(1-\varepsilon _{21}^S)S_1^0. \end{aligned}$$

Substituting these expressions into (A.6), we obtain

A.7 $$\begin{aligned} {\left\{ \begin{array}{ll} (d_1 + m_{21}^S)(S_1^0 - S_{1*})> m_{12}^S(1-\varepsilon _{12}^S)(S_2^0 - S_{2*}), \\ (d_2 + m_{12}^S)(S_2^0 - S_{2*}) > m_{21}^S(1-\varepsilon _{21}^S)(S_1^0 - S_{1*}). \end{array}\right. } \end{aligned}$$

Let $X = S_1^0 - S_{1*}$ and $Y = S_2^0 - S_{2*}$. The inequalities (A.7) implies that

A.8 $$\begin{aligned} (d_1 + m_{21}^S)X> m_{12}^S(1-\varepsilon _{12}^S)Y, \qquad (d_2 + m_{12}^S)Y > m_{21}^S(1-\varepsilon _{21}^S)X. \end{aligned}$$

It is straightforward to see that *X* and *Y* must have the same sign. We now proceed by contradiction to rule out the case where both are negative. Let $X = -u$ and $Y = -v$, where $u >0$ and $v > 0$. Substituting these into (A.8), we have

A.9 $$\begin{aligned} m_{12}^S(1-\varepsilon _{12}^S)v> (d_1 + m_{21}^S)u, \qquad m_{21}^S(1-\varepsilon _{21}^S)u > (d_2 + m_{12}^S)v, \end{aligned}$$

which gives

$$\begin{aligned} m_{12}^S m_{21}^S (1-\varepsilon _{12}^S)(1-\varepsilon _{21}^S) > (d_1 + m_{21}^S)(d_2 + m_{12}^S), \end{aligned}$$

or equivalently,

$$\begin{aligned} m_{12}^S m_{21}^S[ (1-\varepsilon _{12}^S)(1-\varepsilon _{21}^S)-1] > d_1 d_2 + d_1 m_{12}^S + d_2 m_{21}^S, \end{aligned}$$

which is a contradiction, since the left-hand side is non-positive.

Therefore, we obtain $X > 0$ and $Y > 0$, which means $S_1^0 > S_{1*}$ and $S_2^0 > S_{2*}$. By (A.5), we see that $\mathcal {R}_{0,1}^I > 1$ and $\mathcal {R}_{0,2}^I > 1$. This completes the proof. $\square$

## Appendix B Proof of Proposition 2.5

This section focuses on the uniform persistence of strain *I* under the circumstance $m_{12}^{I}>0$ and $m_{21}^{I}>0$ and $\mathcal {R}_0^{J}<1<\mathcal {R}_0^{I}.$

### Proof of Proposition 2.5

We proceed as the proof of (Wang and Zhao 2004, Theorem 2.3). Let

$$\begin{aligned} M^{I}_1&=\begin{bmatrix} \beta ^{I}_1S^0_1-(d_1+\alpha _1^{I}+\gamma _1^{I}+m_{21}^{I})& m^{I}_{12}(1-\varepsilon _{12})\\ m^{I}_{21}(1-\varepsilon _{21})& \beta ^{I}_2S^0_2-(d_2+\alpha _2^{I}+\gamma _2^{I}+m_{12}^{I}) \end{bmatrix} \text{ and } \\ M^{I}_2&=\textrm{diag}(\beta ^{I}_1,\beta ^{I}_2). \end{aligned}$$

Observe that $M^{I}_1=F^{I}-V^{I}$ and that $s(M^{I}_1)>0$ if and only if $\mathcal {R}_0^{I}>1$ by (Driessche and Watmough 2002, Theorem 2). In the case $\mathcal {R}_0^{I}>1,$ choose small $\eta >0$ such that $s(M^{I}_1-\eta M^{I}_2)>0.$ Then $M^{I}_1-\eta M^{I}_2$ is an irreducible Metzler matrix and so $M^{I}_1-\eta M^{I}_2$ has a simple eigenvalue $s(M^{I}_1-\eta M^{I}_2)$ with a positive eigenvector. Consider a perturbed system of (2.1):

B.1 $$\begin{aligned} {\left\{ \begin{array}{ll} S_1'=\Lambda _1-(d_1+\beta ^{I}_1\varepsilon +\beta ^{J}_1\varepsilon )S_1-m_{21}^SS_1+m_{12}^S(1-\varepsilon _{12}^S)S_2\\ S_2'=\Lambda _2-(d_2+\beta ^{I}_2\varepsilon +\beta ^{J}_2\varepsilon )S_2-m_{12}^SS_2+m_{21}^S(1-\varepsilon _{21}^S)S_1 \end{array}\right. } \end{aligned}$$

for small $\varepsilon >0.$ We know that (B.1) admits a globally asymptotically stable positive equilibrium $S^0(\varepsilon ):=(S^0_1(\varepsilon ),S^0_2(\varepsilon ))$ for all small $\varepsilon >0.$ Since $S^0(\varepsilon )$ is continuous in $\varepsilon ,$ we can pick a small $\varepsilon$ such that $S^0(\varepsilon )>S^0(0)-(\eta ,\eta )$. Suppose, to reach a contradiction, that $I_i(t)\le \varepsilon$ for all large *t*. For this $\varepsilon >0,$ since $\mathcal {R}_0^{J}<1$ implies that $\lim \limits _{t\rightarrow \infty }J_i(t)=0$, we see that $J_i(t)<\varepsilon$ for all large $t\gg 1.$ Then

$$\begin{aligned} {\left\{ \begin{array}{ll} S_1'\ge \Lambda _1-(d_1+\beta ^{I}_1\varepsilon +\beta ^{J}_1\varepsilon )S_1-m_{21}^SS_1+m_{12}^S(1-\varepsilon _{12}^S)S_2\\ S_2'\ge \Lambda _2-(d_2+\beta ^{I}_2\varepsilon +\beta ^{J}_2\varepsilon )S_2-m_{12}^SS_2+m_{21}^S(1-\varepsilon _{21}^S)S_1. \end{array}\right. } \end{aligned}$$

for all large $t\gg 1.$ Since $S^0(\varepsilon )$ is globally asymptotically stable in (B.1), we see that $(S_1(t),S_2(t))\ge S^0(0)-(\eta ,\eta )$ for all large *t*. Then

$$\begin{aligned} \begin{bmatrix} I_1'\\ I_2' \end{bmatrix}\ge (M^{I}_1-\eta M^{I}_2)\begin{bmatrix} I_1\\ I_2 \end{bmatrix} \end{aligned}$$

for all large *t*,  which gives $\lim \limits _{t\rightarrow \infty }I_i(t)=\infty ,$ a desired contradiction.

So far, we have shown that at least one of $I_i$ satisfies $\limsup \limits _{t\rightarrow \infty }I_i(t)\ge \varepsilon$. Let

$$\begin{aligned} X&:=\{(S_1,I_1,J_1,S_2,I_2,J_2):S_i\ge 0,I_i\ge 0\},\\ X_0&:=\{(S_1,I_1,J_1,S_2,I_2,J_2)\in X:I_i>0\},\\ \partial X_0&:=X\setminus X_0=\{(S_1,I_1,J_1,S_2,I_2,J_2)\in X:I_1=0 \text{ or } I_2=0\}. \end{aligned}$$

Since $m_{12}^{I}$ and $m_{21}^{I}$ are both positive, we can observe that the $I_1$ and $I_2$ components of every orbit in $\partial X_0$ are both constant zero. Then Proposition 2.3 asserts that every orbit in $\partial X_0$ converges to $E_0$ since $\mathcal {R}_0^{J}<1.$ Clearly, $\{E_0\}$ does not form a cycle in $\partial X_0$. By (Thieme 1993, Theorem 4.6), strain *I* is uniformly persistent with respect to $(X_0,\partial X_0)$. $\square$

## Appendix C Proof of Theorem 1

Now, we are prepared to prove our main results in Section 2.2. First, we give a useful lemma as follows.

### Lemma C.1

Let $k_i>0$, $m_i>0$ and $x_i^*>0$, $i=1,2$, be given such that ${k_1m_1}/{x_1^*}={k_2m_2}/{x_2^*}$. Then

$$\begin{aligned} L(x_1,x_2) {:=} k_1m_1(x_1-x^*_1)\Big (\frac{x_2}{x_1}-\frac{x_2^*}{x_1^*}\Big )+k_2m_2(x_2-x^*_2)\Big (\frac{x_1}{x_2}{-}\frac{x_1^*}{x_2^*}\Big )\le 0 \text{ for } \text{ all } x_1,x_2{>}0.\end{aligned}$$

Furthermore, $L(x_1,x_2)=0$ if and only if $\dfrac{x_1}{x_1^*}=\dfrac{x_2}{x_2^*}.$

### Proof

Let us rewrite *L* as

$$\begin{aligned} L&=k_1m_1(x_1-x^*_1) \frac{x_2x_1^*-x_2x_1+x_2x_1-x_1x_2^*}{x_1x_1^*}\\&\quad +k_2m_2(x_2-x_2^*)\frac{x_1x_2^*-x_1x_2+x_1x_2-x_2x_1^*}{x_2x_2^*}\\&=-\frac{k_1m_1}{x_1^*}\frac{x_2}{x_1}(x_1-x_1^*)^2-\frac{k_2m_2}{x_2^*}\frac{x_1}{x_2}(x_2-x_2^*)^2+(\frac{k_1m_1}{x_1^*}+\frac{k_2m_2}{x_2^*})(x_1-x^*_1)(x_2-x_2^*)\\&=-\frac{k_1m_1}{x_1^*}\Big [\sqrt{\frac{x_2}{x_1}}(x_1-x^*_1)-\sqrt{\frac{x_1}{x_2}}(x_2-x_2^*)\Big ]^2\le 0. \end{aligned}$$

Clearly, $L(x_1,x_2)=0$ if and only if

$$\begin{aligned} \sqrt{\frac{x_2}{x_1}}(x_1-x^*_1)=\sqrt{\frac{x_1}{x_2}}(x_2-x_2^*), \end{aligned}$$

which is equivalent to $\dfrac{x_1}{x_1^*}=\dfrac{x_2}{x_2^*}$. This completes the proof. $\square$

With this lemma at hand, we establish the global stability of the strict strain-*I* endemic equilibrium $E_{1}$.

### Proof of Theorem 1

Our proof is inspired by the argument used in Lou and Wu (2011) by using a powerful Lyapunov function reported in Goh (1977); Hsu (1978). We define the Lyapunov function

$$\begin{aligned} L(X):=\sum _{i=1}^2(k_iL_i^S+\ell _iL_i^{I}+p_iJ_i), \end{aligned}$$

where $k_i,\ell _i,p_i$ are nonnegative constants to be determined and

C.1 $$\begin{aligned} L_i^S(X):=S_i-S_{i*}-S_{i*}\ln \dfrac{S_i}{S_{i*}},\quad L_i^{I}(X):=I_i-I_{i*}-I_{i*}\ln \dfrac{I_i}{I_{i*}}. \end{aligned}$$

By the equation satisfied by $S_i$ and using the identity

$$\begin{aligned} 0=\dfrac{\Lambda _i}{S_{i*}}-\beta _i^II_{i*}-(d_i+m_{ji}^S)+m_{ij}^S(1-\varepsilon _{ij}^S)\frac{S_{j*}}{S_{i*}}, \end{aligned}$$

one has

C.2 $$\begin{aligned} ({L}_i^S)'(X)&=(S_i-S_{i*})\Big [\dfrac{\Lambda _i}{S_i}-\beta ^I_iI_i-\beta ^J_iJ_i -(d_i+m_{ji}^S)+m_{ij}^S(1-\varepsilon _{ij}^S)\frac{S_j}{S_i}\nonumber \\&\hspace{4.2em}-\Big (\dfrac{\Lambda _i}{S_{i*}}-\beta _i^II_{i*}-(d_i+m_{ji}^S)+m_{ij}^S(1-\varepsilon _{ij}^S)\frac{S_{j*}}{S_{i*}}\Big )\Big ]\nonumber \\&=\Lambda _i(S_i-S_{i*})\Big (\dfrac{1}{S_i}-\dfrac{1}{S_{i*}}\Big )+m_{ij}^S(1-\varepsilon _{ij}^S)(S_i-S_{i*})\Big (\frac{S_j}{S_i}-\frac{S_{j*}}{S_{i*}}\Big )\nonumber \\&\hspace{4.2em}-(S_i-S_{i*})[\beta ^{I}_i(I_i-I_{i*})+\beta ^{J}_iJ_i]. \end{aligned}$$

Also, by the equation satisfied by $I_i$ and using the identity

C.3 $$\begin{aligned} \beta ^{I}_iS_{i*}-(d_i+\alpha ^{I}_i+\gamma ^{I}_i)-m_{ji}^{I}+m_{ij}^{I}(1-\varepsilon _{ij})\frac{I_{j*}}{I_{i*}}=0, \end{aligned}$$

we have

C.4 $$\begin{aligned} (L_i^{I})'(X)&=m_{ij}^{I}(1-\varepsilon _{ij})(I_i-I_{i*})\Big (\frac{I_j}{I_i}-\frac{I_{j*}}{I_{i*}}\Big )+\beta ^{I}_i(S_i-S_{i*})(I_i-I_{i*}). \end{aligned}$$

On the other hand, multiplying (C.3) by $J_i$ yields that

C.5 $$\begin{aligned} \beta ^{I}_i S_{i*} J_i-(d_i+\alpha ^{I}_i+\gamma ^{I}_i+m_{ji}^{I})J_i+m_{ij}^{I}(1-\varepsilon _{ij})\frac{I_{j*}}{I_{i*}}J_i=0. \end{aligned}$$

We then subtract (C.5) from

$$\begin{aligned}J_i'=\beta _i^{J}S_iJ_i-(d_i+\alpha _i^{J}+\gamma _i^{J}+m_{ji}^{J})J_i+m_{ij}^{J}(1-\varepsilon _{ij})J_j\end{aligned}$$

to conclude that

$$\begin{aligned} J_i'&=(\beta _i^{J}S_i- \beta ^{I}_i S_{i*})J_i+\big ((\alpha ^{I}_i+\gamma ^{I}_i)-(\alpha _i^{J}+\gamma _i^{J})\big )J_i\\&\hspace{4.2em}+\Big (m_{ji}^{I}-m_{ji}^{J}-m_{ij}^{I}(1-\varepsilon _{ij})\frac{I_{j*}}{I_{i*}}\Big )J_i+m_{ij}^{J}(1-\varepsilon _{ij})J_j.\\&=\beta _i^{J}(S_i-S_{i*})J_i+(\beta _i^{J}-\beta ^{I}_i)S_{i*}J_i+\big ((\alpha ^{I}_i+\gamma ^{I}_i)-(\alpha _i^{J}+\gamma _i^{J})\big )J_i\\&\hspace{4.2em}+\Big (m_{ji}^{I}-m_{ji}^{J}-m_{ij}^{I}(1-\varepsilon _{ij})\frac{I_{j*}}{I_{i*}}\Big )J_i+m_{ij}^{J}(1-\varepsilon _{ij})J_j. \end{aligned}$$

In view of **(H1)** and definition of $m_*$, we have

C.6 $$\begin{aligned} \sum _{i=1}^2p_iJ_i'&\le p_1\beta _1^J(S_1-S_{1*})J_1 +p_2\beta _2^J(S_2-S_{2*})J_2 \nonumber \\&\hspace{1em}+J_1[p_1(m_{21}^{I}-m_{21}^{J}-m_*)+p_2m_{21}^{J}(1-\varepsilon _{21})]\nonumber \\&\hspace{1em}+J_2\Big [p_1m_{12}^{J}(1-\varepsilon _{12})+p_2(m^{I}_{12}-m_{12}^{J}-m_{21}^{I}(1-\varepsilon _{21})\frac{I_{1*}}{I_{2*}})\Big ]. \end{aligned}$$

Combining (C.2), (C.4) and (C.6), we obtain

$$\begin{aligned} L'(X)=\sum _{i=1}^2(k_i(L_i^S)'+\ell _i (L_i^{I})'+p_iJ_i':=\sum _{j=1}^6 W_j, \end{aligned}$$

where

$$\begin{aligned} W_1=&-\sum _{i=1}^2k_i\Lambda _i\frac{1}{S_iS_{i*}}(S_i-S_{i*})^2\le 0,\\ W_2=&\sum _{i=1,i\ne j}^2k_im_{ij}^S(1-\varepsilon _{ij}^S)(S_i-S_{i*})\Big (\frac{S_j}{S_i}-\frac{S_{j*}}{S_{i*}}\Big ),\\ W_3=&\sum _{i=1,i\ne j}^2\ell _im_{ij}^{I}(1-\varepsilon _{ij})(I_i-I_{i*})\Big (\frac{I_j}{I_i}-\frac{I_{j*}}{I_{i*}}\Big ),\\ W_4=&J_1[p_1(m_{21}^{I}-m_{21}^{J}-m_*)+p_2m_{21}^{J}(1-\varepsilon _{21})] \\&\hspace{3em}+J_2\Big [p_1m_{12}^{J}(1-\varepsilon _{12})+p_2(m^{I}_{12}-m_{12}^{J}-m_{21}^{I}(1-\varepsilon _{21})\frac{I_{1*}}{I_{2*}})\Big ],\\ W_5=&\sum _{i=1}^2\Big [(\ell _i-k_i)\beta ^{I}_i(S_i-S_{i*})(I_i-I_{i*}) +(p_i-k_i)\beta ^{{J}}_i(S_i-S_{i*})J_i\Big ], \\ W_6=&\sum _{i=1}^2J_i\Big [(\beta _i^{J}-\beta ^{I}_i)S_{i*}+\big ((\alpha ^{I}_i+\gamma ^{I}_i)-(\alpha _i^{J}+\gamma _i^{J})\big )\Big ]. \end{aligned}$$

Now we will choose suitable $k_i,\ell _i,p_i\ge 0$ ($i=1,2$) such that $L'(X)\le 0$. First, we pick

C.7 $$\begin{aligned} k_1=\ell _1=p_1=1. \end{aligned}$$

Then we obtain

C.8 $$\begin{aligned} W_5=(\ell _2-k_2)\beta _2^{I} (S_2-S_{2*})(I_2-I_{2*}) +(p_2-k_2)\beta _2^{{J}}(S_2-S_{2*})J_2. \end{aligned}$$

To confirm the sign of $W_5$, we divide our discussion into two parts: $\lambda >0$ or $\lambda =0$, where $\lambda$ is given in (2.9).

We first assume that $\lambda >0$. Since $m_{12}^{I}>0$ and $m_{21}^{I}>0$, by (2.9), we have $m_{12}^S>0$ and $m_{21}^S>0$. Hence, we can choose $k_2,\ell _2,p_2>0$ such that

$$\begin{aligned} \frac{m_{12}^S(1-\varepsilon _{12}^S)}{S_{1*}}&=\frac{k_2m_{21}^S(1-\varepsilon _{21}^S)}{S_{2*}},\\ \frac{m^{I}_{12}(1-\varepsilon _{12})}{I_{1*}}&=\frac{\ell _2m_{21}^{I}(1-\varepsilon _{21})}{I_{2*}},\quad \frac{m^{I}_{12}(1-\varepsilon _{12})}{I_{1*}}=\frac{p_2m_{21}^{I}(1-\varepsilon _{21})}{I_{2*}}, \end{aligned}$$

or equivalently,

C.9 $$\begin{aligned} k_2=\dfrac{m_{12}^S(1-\varepsilon _{12}^S)}{m_{21}^S(1-\varepsilon _{21}^S)}\dfrac{S_{2*}}{S_{1*}}>0,\quad \ell _2=p_2=\dfrac{m^{I}_{12}(1-\varepsilon _{12})}{m_{21}^{I}(1-\varepsilon _{21})}\dfrac{I_{2*}}{I_{1*}}=\frac{m_*}{m_{21}^{I}(1-\varepsilon _{21})}>0. \end{aligned}$$

In view of (C.9) and (2.9), we have $k_2=\ell _2=p_2$. Together with (C.8) we see that $W_5\equiv 0$.

If $\lambda =0$, we can choose

$$\begin{aligned} k_2=\ell _2=p_2=\dfrac{m_*}{m_{21}^{I}(1-\varepsilon _{21})}, \end{aligned}$$

which implies that $W_5\equiv 0$.

With the coefficients properly chosen and $W_5$ eliminated, we now consider the remaining terms. By **(H1)**, we have $W_6\le 0$. We next consider $W_2$. By assumption **(M0)**, if $m_{12}^S=m_{21}^S=0$, it is clear to see that $W_2\equiv 0$. If $m_{12}^S>0$ and $m_{21}^S>0$, thanks to Lemma C.1, we see that $W_2\le 0$ with $W_2=0$ if and only if $S_1/S_{1*}=S_2/S_{2*}$. Similarly, by virtue of **(M1)**, we can apply Lemma C.1 to conclude that $W_3\le 0$, where $W_3=0$ if and only if $I_1/I_{1*}=I_2/I_{2*}$.

Next, we focus on $W_4$. We claim that

C.10 $$\begin{aligned}&p_1(m_{21}^{I}-m_{21}^{J}-m_*)+p_2m_{21}^{J}(1-\varepsilon _{21})\le 0,\end{aligned}$$

C.11 $$\begin{aligned}&p_1m_{12}^{J}(1-\varepsilon _{12})+p_2\Big (m^{I}_{12}-m_{12}^{J}-m_{21}^{I}(1-\varepsilon _{21})\frac{I_{1*}}{I_{2*}}\Big )\le 0 \end{aligned}$$

By the choice of $p_i$, (C.7) and (C.9), we see that (C.10) is equivalent to

$$\begin{aligned}(m_{21}^{J}-m_{21}^{I})(m_*-m_{21}^{I})\le 0\end{aligned}$$

and that (C.11) is equivalent to

$$\begin{aligned}(m_{12}^{J}-m^{I}_{12})(m_{**}-m_{21}^{I})\ge 0.\end{aligned}$$

Then one can easily check that both these two inequalities hold if and only if one of (1)–(3) holds.

From the above discussions, we obtain that $L'\le 0$ provided that (C.7) and (C.9) holds. Moreover, $L'=0$ if and only if $W_i=0$ for each $i=1,2,\ldots ,6$. That is,

C.12 $$\begin{aligned} S_i=S_{i*},\ i=1,2,\quad \dfrac{S_1}{S_{1*}}=\dfrac{S_2}{S_{2*}},\quad \dfrac{I_1}{I_{1*}}=\dfrac{I_2}{I_{2*}}. \end{aligned}$$

Let $\mathcal {M}$ be the largest invariant set in $\{X\in \Gamma :L'(X)=0\}$ with respect to the flow of (1.3). Let $X(t)=(S_1(t),I_1(t),J_1(t),S_2(t),I_2(t),J_2(t))$ be a solution of (1.3) and belong to $\mathcal {M}$. It follows that $S_i(t)=S_{i*}$ for all $t\ge 0$ and $i=1,2$. On the other hand, there is a differentiable function $\eta :[0,\infty )\rightarrow (0,\infty )$ such that for all $t\ge 0,$

C.13 $$\begin{aligned} \dfrac{I_1(t)}{I_{1*}}=\dfrac{I_2(t)}{I_{2*}}=\eta (t)>0 \text{ for } \text{ all } t\ge 0. \end{aligned}$$

It follows that

$$\begin{aligned} {\left\{ \begin{array}{ll} I_1'=\big [\big (\beta ^{I}_1S_{1*}-(d_1+\alpha _1^{I}+\gamma _1^{I}+m_{21}^{I})\big )I_{1*}+m_{12}^{I}(1-\varepsilon _{12})I_{2*}\big ]\eta (t)=0\ \forall t\ge 0,\\ I_2'=\big [\big (\beta ^{I}_2S_{2*}-(d_2+\alpha _2^{I}+\gamma _2^{I}+m_{12}^{I})\big )I_{2*}+m_{21}^{I}(1-\varepsilon _{21})I_{1*}\big ]\eta (t)=0\ \forall t\ge 0. \end{array}\right. } \end{aligned}$$

This means that $I_1(t),I_2(t)$ are constant functions.

Next, in view of (2.9) and (Li and Shuai 2009, Theorem 4.1), we obtain that the system (1.3) with $J_i\equiv 0$ has a unique positive equilibrium. This implies that $E_{1}$ is the unique strict strain-*I* endemic equilibrium. By the uniqueness of $E_{1}$, (C.13) and the equilibrium equations for $I_1,I_2$, we have $I_i(t)=I_{i*}$ for all $t\ge 0,$ for each $i=1,2$. Finally, using that $S_i(t)=S_{i*}$ for all $t\ge 0$, from the equation of $S_i$ we see that

$$\begin{aligned} {\left\{ \begin{array}{ll} 0=\Lambda _1-\beta ^{I}_1S_{1*}I_{1*}-\beta ^{J}_1S_{1*}J_1(t)-d_1S_{1*}-m_{21}^SS_{1*}+m_{12}^S(1-\varepsilon _{12}^S)S_{2*},\\ 0=\Lambda _1-\beta ^{I}_2S_{2*}I_{2*}-\beta ^{J}_2S_{2*}J_1(t)-d_2S_{2*}-m_{12}^SS_{2*}+m_{21}^S(1-\varepsilon _{21}^S)S_{1*}. \end{array}\right. } \end{aligned}$$

By the definition of $E_{1}$, we see that $J_1(t)=J_2(t)=0$ for all $t\ge 0.$ Therefore, $\mathcal {M}$ is the singleton $\{E_{1}\}.$ Finally $L(X)\rightarrow \infty$ as $S_i\rightarrow 0$ or $I_i\rightarrow 0$ for some $i=1,2$. By LaSalle’s Invariance Principle (see (LaSalle 1960)), $E_{1}$ is globally asymptotically stable in $\textrm{int}(\Gamma )$. This completes the proof. $\square$

### Remark C.1

If the inequality in **(H1)** is strict, the uniqueness of $E_{1}$ is not required in the proof of Theorem 1. In fact, we can see from the proof of that $\mathcal {M}=\{E_{1}\}$ can be obtained much easier when the inequality in **(H1)** is strict, because the set $\{X\in \Gamma :L'(X)=0\}$ was already the singleton $\{E_{1}\}$.

## Appendix D Proofs of Theorem 2 and Theorem 3

This section is devoted to dealing with the case $m_{12}^{I}=m_{21}^{I}=0.$ First, we show that $(m_{12}^{I},m_{21}^{I})=(0,0)$ will be a migration strategy for strain *I* to outcompete strain *J* in both patches under the assumption (2.11).

### Proof of Theorem 2

In the proof of Theorem 1, $W_3\equiv 0$ since $m_{12}^{I}=m_{21}^{I}=0$. Also, **(H1)** asserts that $W_6\le 0.$ By **(M0)**, it suffices to consider the following two cases of $m_{ij}^S:$

1. both $m_{12}^S$ and $m_{21}^S$ are positive.
2. both $m_{12}^S$ and $m_{21}^S$ are zero.

In the case (1), we take

$$\begin{aligned} k_1=\ell _1=p_1=1,~ k_2=\ell _2=p_2=\dfrac{m_{12}^S(1-\varepsilon _{12}^S)}{m_{21}^S(1-\varepsilon _{21}^S)}\dfrac{S_{2*}}{S_{1*}}. \end{aligned}$$

Then $W_1=W_2=0$ if and only if $S_i=S_{i*}$ for each $i=1,2$. Also, $W_5\equiv 0$ in this case. Moreover, by (2.11) we have

$$\begin{aligned} &  W_4=J_1m_{21}^{J}\Big (\dfrac{m_{12}^S(1-\varepsilon _{12}^S)S_{2*}}{m_{21}^S(1-\varepsilon _{21}^S)S_{1*}}(1-\varepsilon _{21})-1\Big ) \\ &  +J_2m_{12}^{J}(1-\varepsilon _{12})\Big (1-\dfrac{m_{12}^S(1-\varepsilon _{12}^S)S_{2*}}{m_{21}^S(1-\varepsilon _{21}^S)S_{1*}}\dfrac{1}{1-\varepsilon _{12}}\Big )\le 0.\end{aligned}$$

Let $\mathcal {M}$ be the largest invariant set in $\{X\in \Gamma :L'(X)=0\}$ with respect to the flow of (1.3). Let $X(t)=(S_1(t),I_1(t),J_1(t),S_2(t),I_2(t),J_2(t))$ be a solution of (1.3) and belong to $\mathcal {M}$. Then $S_i(t)=S_{i*}$ for all $t\ge 0$ and $i=1,2.$ From (1.3), *X*(*t*) satisfies

$$\begin{aligned} {\left\{ \begin{array}{ll} 0=\Lambda _1-\beta ^{I}_1S_{1*}I_{1}(t)-\beta ^{J}_1S_{1*}J_{1}(t)-d_1S_{1*}-m_{21}^SS_{1*}+m_{12}^S(1-\varepsilon _{12}^S)S_{2*},\\ J_1'(t)=\beta ^{J}_1S_{1*}J_1(t)-(d_1+\alpha _1^{J}+\gamma _1^{J})J_1(t)-m_{21}^{J}J_1(t)+m_{12}^{J}(1-\varepsilon _{12})J_2(t),\\ 0=\Lambda _1-\beta ^{I}_2S_{2*}I_{2}(t)-\beta ^{J}_1S_{2*}J_{2}(t)-d_2S_{2*}-m_{12}^SS_{2*}+m_{21}^S(1-\varepsilon _{21}^S)S_{1*}\\ J_2'(t)=\beta ^{J}_2S_{2*}J_2(t)-(d_2+\alpha _2^{J}+\gamma _2^{J})J_2(t)-m_{12}^{J}J_2(t)+m_{21}^{J}(1-\varepsilon _{21})J_1(t). \end{array}\right. } \end{aligned}$$

Since $\varepsilon _{ij},m_{ij}^S$ are all nonzero, the inequalities in (2.11) cannot both be equality. Thus, if $W_4=0$, then at least one of $J_1$ and $J_2$ is zero. This implies that at least one of $J_1(t)$ and $J_2(t)$ is a zero function for $t\ge 0,$ say $J_1(t)=0$ for all $t\ge 0.$ Then the equation of $J_1$ shows that

$$\begin{aligned} 0=m_{12}^{J}(1-\varepsilon _{12})J_2(t),~t\ge 0, \end{aligned}$$

which shows that $J_2(t)=0$ for all $t\ge 0,$ since $m_{12}^J>0$ (by **(M2)**) and $\varepsilon _{12}<1$. Therefore, for all $t\ge 0,$ we have

$$\begin{aligned} {\left\{ \begin{array}{ll} 0=\Lambda _1-\beta ^{I}_1S_{1*}I_{1}(t)-d_1S_{1*}-m_{21}^SS_{1*}+m_{12}^S(1-\varepsilon _{12}^S)S_{2*},\\ 0=\Lambda _1-\beta ^{I}_2S_{2*}I_{2}(t)-d_2S_{2*}-m_{12}^SS_{2*}+m_{21}^S(1-\varepsilon _{21}^S)S_{1*}. \end{array}\right. } \end{aligned}$$

By the definition of $E_{1},$ we have $I_i(t)=I_{i*}$ for all $t\ge 0$ and $i=1,2.$ Therefore, $\mathcal {M}=\{E_{1}\}$ Then we conclude by LaSalle’s Invariance Principle that $E_{1}$ is globally asymptotically stable in $\textrm{int}(\Gamma )$.

In the case (2), we take $k_i=\ell _i=p_i=1$ for each $i=1,2.$ Then $W_2=W_3=W_5\equiv 0$, and $W_4=-\varepsilon _{21}{m_{21}^J}J_1-\varepsilon _{12}{m_{12}^J}J_2.$ Since $\varepsilon _{21}$ and $\varepsilon _{12}$ are nonzero, we have $W_4=0$ if and only if $J_1=J_2=0.$ Then similarly as in the previous paragraph, we see that $\mathcal {M}=\{E_{1}\}$ and so $E_{1}$ is globally asymptotically stable in $\textrm{int}(\Gamma ).$ The proof is now complete. $\square$

### Remark D.1

If the inequality in **(H1)** is strict, it can be checked that the conclusion of Theorem 2 still holds if $\varepsilon _{ij}^I=0$ or $m_{ij}^{J}=0$ for some $i,j\in \{1,2\}$ with $i\ne j$.

Next, we show that the dynamics is determined by the local reproduction numbers whenever all the infected classes do not migrate.

### Proof of Theorem 3

In view of Remark 2.4, we see that (b) and (c) imply the strict inequality in **(H1)**. By **(M0)**, it suffices to consider the following two cases of $m_{ij}^S:$

1. both $m_{12}^S$ and $m_{21}^S$ are positive.
2. both $m_{12}^S$ and $m_{21}^S$ are zero.

Suppose that $m_{12}^S$ and $m_{21}^S$ are both positive. Recall that **(M3)** indicates that $m_{12}^I=m_{21}^I=m_{12}^J=m_{21}^J=0.$ Then, following the proof of Theorem 1 with the same Lyapunov function *L*, where

$$\begin{aligned} k_1=\ell _1=p_1=1,\quad k_2=\ell _2=p_2=\dfrac{m_{12}^S(1-\varepsilon _{12}^S)}{m_{21}^S(1-\varepsilon _{21}^S)}\dfrac{S_{2*}}{S_{1*}}. \end{aligned}$$

We can deduce $L'=W_1+W_2+W_6\le 0$. Moreover, $L'=0$ if and only if $S_i=S_{i*}$ and $J_i=0$ for each $i=1,2$ (since the inequality in **(H1)** is strict). In view of Remark C.1, we see that $\mathcal {M}=\{E_{1}\}.$ We now conclude again by LaSalle’s Invariance Principle that $E_{1}$ is globally asymptotically stable in $\textrm{int}(\Gamma )$.

On the other hand, if $m_{12}^S=m_{21}^S=m_{12}^{I}=m_{21}^{I}=m_{12}^J=m_{21}^J=0,$ then $L'=W_1+W_6$ when we choose $k_1=k_2=\ell _1=\ell _2=p_1=p_2=1.$ Again $L'=0$ if and only if $S_i=S_{i*},J_i=0$ for each $i=1,2$ and similarly as in the previous paragraph, $E_{1}$ is globally asymptotically stable in $\textrm{int}(\Gamma )$. $\square$

## Appendix E Proofs of Proposition 2.6 and Theorem 4

We finally prove the existence and the global stability of a coexistence endemic equilibrium $E_{3}$ in this section. Below we deal with the existence first.

### Proof of Proposition 2.6

For the later use, let

$$\begin{aligned}&\overline{M^{k}}=\begin{bmatrix} \beta ^{k}_1S_{1*}^{\ell }-(d_1+\alpha _1^{k}+\gamma _1^{k}+m_{21}^{k})& m^{k}_{12}(1-\varepsilon _{12})\\ m^{k}_{21}(1-\varepsilon _{21})& \beta ^{k}_2S_{2*}^{\ell }-(d_2+\alpha _2^{k}+\gamma _2^{k}+m_{12}^{k}) \end{bmatrix},\\&\quad \quad k,\ell \in \{I,J\},\quad k\ne \ell , \end{aligned}$$

where $$S_{i*}^I:=S_{i*}.$$ Set

$$\begin{aligned} X&:=\{(S_1,I_1,J_1,S_2,I_2,J_2):S_i\ge 0,I_i\ge 0,J_i\ge 0\},\\X_0&:=\{(S_1,I_1,J_1,S_2,I_2,J_2)\in X:I_i>0,J_i>0\} \end{aligned}$$

and $\partial X_0:=X\setminus X_0$. We first show that (1.3) is uniformly persistent with respect to $(X_0,\partial X_0)$. Rewrite the first and fifth equations in (1.3) as

$$\begin{aligned} {\left\{ \begin{array}{ll} S_1'=\Lambda _1-\beta ^{I}_1S_1I_1-\beta ^{J}_1S_1({J_1}-J_{1*})-d_1S_1-(m_{21}^S+\beta ^{J}_1J_{1*})S_1+m_{12}^S(1-\varepsilon _{12}^S)S_2\\ S_2'=\Lambda _2-\beta ^{I}_2S_2I_2-\beta _2^{J}S_2({J_2}-J_{2*})-d_2S_2-(m_{12}^S+\beta _2^{J}J_{2*})S_2+m_{21}^S(1-\varepsilon _{21}^S)S_1. \end{array}\right. } \end{aligned}$$

Note that the perturbed system

$$\begin{aligned} {\left\{ \begin{array}{ll} S_1'=\Lambda _1-((\beta ^{I}_1+\beta ^{J}_1)\varepsilon +d_1)S_1-(m_{21}^S+\beta ^{J}_1J_{1*})S_1+m_{12}^S(1-\varepsilon _{12}^S)S_2\\ S_2'=\Lambda _2-((\beta ^{I}_2+\beta ^{J}_2)\varepsilon +d_2)S_2-(m_{12}^S+\beta _2^{J}J_{2*})S_2+m_{21}^S(1-\varepsilon _{21}^S)S_1 \end{array}\right. } \end{aligned}$$

has a globally asymptotically stable positive equilibrium $S^*(\varepsilon ):=(S^*_1(\varepsilon ),S^*_2(\varepsilon ))$ for all small $\varepsilon .$ Pick a small $\varepsilon$ such that $S^*(\varepsilon )>S^*(0)-(\eta ,\eta )$, where $\eta >0$ was chosen such that $s(\overline{M^{I}}-\eta M^{I}_2)>0$. The choice of $\eta$ uses the assumption $\overline{\mathcal {R}_0^{I}}>1.$ If $\max \{I_1(t),I_2(t),|J_1(t)-J_{1*}|,|J_2(t)-J_{2*}|\}\le \varepsilon$ for all large *t*, then

$$\begin{aligned} {\left\{ \begin{array}{ll} S_1'\ge \Lambda _1-((\beta ^{I}_1+\beta ^{J}_1)\varepsilon +d_1)S_1-(m_{21}^S+\beta ^{J}_1J_{1*})S_1+m_{12}^S(1-\varepsilon _{12}^S)S_2\\ S_2'\ge \Lambda _2-((\beta ^{I}_2+\beta ^{J}_2)\varepsilon +d_2)S_2-(m_{12}^S+\beta _2^{J}J_{2*})S_2+m_{21}^S(1-\varepsilon _{21}^S)S_1 \end{array}\right. } \end{aligned}$$

and so $(S_1(t),S_2(t))\ge S^*(0)-(\eta ,\eta )$ for all large *t*. Then

$$\begin{aligned} \begin{bmatrix} I_1'\\ I_2' \end{bmatrix}\ge (\overline{M^{I}}-\eta M^{I}_2)\begin{bmatrix} I_1\\ I_2 \end{bmatrix} \end{aligned}$$

for all large *t*,  where $M_2^I=\textrm{diag}(\beta _1^I,\beta _2^I)$. By **(M1)**, $\overline{M^{I}}-\eta M^{I}_2$ is an irreducible Metzler matrix, which implies that it has a positive eigenvector corresponding to a simple eigenvalue $s(\overline{M^{I}}-\eta M^{I}_2)$, which gives $\lim \limits _{t\rightarrow \infty }I_i(t)=\infty ,$ a contradiction. Thus,

$$\begin{aligned} \limsup \limits _{t\rightarrow \infty }\max \{I_1(t),I_2(t),|J_1(t)-J_{1*}|,|J_2(t)-J_{2*}|\}\ge \varepsilon , \end{aligned}$$

Similarly,

$$\begin{aligned} \limsup \limits _{t\rightarrow \infty }\max \{|I_1(t)-I_{1*}|,|I_2(t)-I_{2*}|,J_1(t),J_2(t)\}\ge \varepsilon . \end{aligned}$$

Note that (c) implies that every orbit in $\partial X_0$ converges to one of $E_0,E_{1},E_{2}$. By (a) and (Li and Shuai 2009, Proposition 3.2), $E_0$ repels every orbit in $\partial X_0$ near $E_0$ and so $E_0$ cannot be chained to itself in $\partial X_0$. (c) also implies that $E_{1},E_{2}$ cannot be chained to themselves. Furthermore, since $\{X\in \Gamma :I_1=I_2=0\}$ and $\{X\in \Gamma :J_1=J_2=0\}$ are both positively invariant, we see that $E_0,E_{1},E_{2}$ cannot form a cycle. Therefore, we can conclude that (1.3) is uniformly persistent with respect to $(X_0,\partial X_0)$. Note that (1.3) is point dissipative (as in Proposition 2.1). By (Zhao 1995, Theorem 2.4), a coexistence endemic equilibrium $E_{3}$ exists. $\square$

Next, the global stability of $E_{3}$ is established as follows. We give the following graph-theoretical lemma, which is extracted from the arguments in Li and Shuai (2009).

### Lemma E.1

Let $n\ge 2$ be a fixed positive integer. Let $m_{ij}^{k}>0$ for $i,j=1,\ldots ,n,i\ne j,k=1,\ldots ,m,$ and $m_{ii}^{k}=0$ for $i=1,\ldots ,n.$ Let $x_{i*}^k>0$ for $i=1,\ldots ,n,k=1,\ldots ,m.$ Let $(\mathcal {G},W)$ be a directed graph with weight matrix $W=[w_{ij}],$ where $w_{ij}=m_{ij}^1x_{i*}^1.$ Let

E.1 $$\begin{aligned} c_i=\sum _{\mathcal {T}\in \mathbb {T}_i}w(\mathcal {T}), \end{aligned}$$

where $\mathbb {T}_i$ is the set of all spanning trees of $(\mathcal {G},W)$ rooted at *i*,  and $w(\mathcal {T})$ is the weight of a tree $\mathcal {T}$ defined by the product of the weights on all its arcs. If there is a $\lambda >0$ such that

E.2 $$\begin{aligned} \lambda m_{ij}^1x_{i*}^1=m_{ij}^kx_{i*}^k \text{ for } k=2,\ldots ,m,1\le i,j\le n, \end{aligned}$$

then

E.3 $$\begin{aligned} \sum _{i=1}^nc_i\sum _{k=1}^m\sum _{j=1}^nm_{ij}^kx_{i*}^k\Big (\dfrac{x_j^k}{x_{j*}^k}+\ln \dfrac{x_{j*}^k}{x_j^k}-\dfrac{x_i^k}{x_{i*}^k}-\ln \dfrac{x_{i*}^k}{x_i^k}\Big )=0 \end{aligned}$$

for all $$x_i^k>0,i=1,\ldots ,n,k=1,\ldots ,m.$$

### Proof

By (E.2), we rewrite the left-hand side of (E.3) as

$$\begin{aligned} \sum _{i=1}^nc_i\sum _{j=1}^nm_{ij}^1x_{i*}^1(G_j(x_j^1,\ldots ,x_j^k)-G_i(x_i^1,\ldots ,x_i^k)), \end{aligned}$$

where

$$\begin{aligned} G_i(x_i^1,\ldots ,x_i^k)=\Big (\dfrac{x_i^1}{x_{i*}^1}+\ln \dfrac{x_{i*}^1}{x_i^1}\Big )+\lambda \sum _{k=2}^m\Big (\dfrac{x_j^k}{x_{j*}^k}+\ln \dfrac{x_{j*}^k}{x_j^k}\Big ). \end{aligned}$$

By (Li and Shuai 2010, Theorem 2.3), the left-hand side of (E.3) equals 0, which proves this lemma. $\square$

### Proof of Theorem 4

We proceed as in the proof of (Li and Shuai 2009, Theorem 4.1). We consider the case that $m_{12}^S>0$ and $m_{21}^S>0$. Let $c_i$ ($i=1,2$) be given by (E.1) for $w_{ij}=m_{ij}^S(1-\varepsilon _{ij}^S)S_{i\Diamond }$. Consider the Lyapunov function

$$\begin{aligned}L:=\sum _{i=1}^2c_i(L_i^S+L_i^{I}+L_i^{J}),\end{aligned}$$

where

$$\begin{aligned} &  L_i^S:=S_i-S_{i\Diamond }-S_{i\Diamond }\ln ({S_i}/{S_{i\Diamond }}),~ L_i^{I}:=I_i-I_{i\Diamond }-I_{i\Diamond }\ln (I_i/I_{i\Diamond }),\\ &  \quad ~L_i^{J}:=J_i-J_{i\Diamond }-J_{i\Diamond }\ln (J_i/J_{i\Diamond }).\end{aligned}$$

We still treat $m_{ii}^S=m_{ii}^I=m_{ii}^J=0$ for each $i=1,2.$ By Lemma E.1, we have

$$\begin{aligned} \sum _{i=1}^2c_i\sum _{j=1 }^2\bigg (&m_{ij}^S(1-\varepsilon _{ij}^S)S_{j\Diamond }\bigg (\dfrac{S_j}{S_{j\Diamond }}+\ln \dfrac{S_{j\Diamond }}{S_j}-\dfrac{S_i}{S_{i\Diamond }}-\ln \dfrac{S_{i\Diamond }}{S_i}\bigg )\\ +&m_{ij}^I(1-\varepsilon _{ij})I_{j\Diamond }\bigg (\dfrac{I_j}{I_{j\Diamond }}+\ln \dfrac{I_{j\Diamond }}{I_j}-\dfrac{I_i}{I_{i\Diamond }}-\ln \dfrac{I_{i\Diamond }}{I_i}\bigg )\\ +&m_{ij}^J(1-\varepsilon _{ij})J_{j\Diamond }\bigg (\dfrac{J_j}{J_{j\Diamond }}+\ln \dfrac{J_{j\Diamond }}{J_j}-\dfrac{J_i}{J_{i\Diamond }}-\ln \dfrac{J_{i\Diamond }}{J_i}\bigg )\bigg )=0. \end{aligned}$$

Then

$$\begin{aligned} L'=&\sum _{i=1}^2c_i\Lambda _i(S_i-S_{i\Diamond })(\dfrac{1}{S_i}\\&-\dfrac{1}{S_{i\Diamond }})+\sum _{i=1}^2c_i\sum _{j=1 }^2m_{ij}^S(1-\varepsilon _{ij}^S)S_{j\Diamond }\bigg (1-\dfrac{S_{i\Diamond }S_j}{S_iS_{j\Diamond }}+\ln \dfrac{S_{i\Diamond } S_j}{S_iS_{j\Diamond }}\bigg )\\&+\sum _{i=1}^2c_i\left( \sum _{j=1 }^2m^{I}_{ij}(1-\varepsilon _{ij})I_{j\Diamond }\bigg (1-\dfrac{I_{i\Diamond } I_j}{I_iI_{j\Diamond }}+\ln \dfrac{I_{i\Diamond } I_j}{I_iI_{j\Diamond }}\bigg )\right. \\&\quad \left. + \sum _{j=1 }^2m^{J}_{ij}(1-\varepsilon _{ij})J_{j\Diamond }\bigg (1-\dfrac{J_{i\Diamond } J_j}{J_iJ_{j\Diamond }}+\ln \dfrac{J_{i\Diamond }J_j}{J_iJ_{j\Diamond }}\bigg )\right) . \end{aligned}$$

Clearly, $1-x+\ln x\le 0$ and the equality holds if and only if $x=1.$ Also note that $(S_i-S_{i\Diamond })(\dfrac{1}{S_i}-\dfrac{1}{S_{i\Diamond }})\le 0$ and the equality holds if and only if $S_i=S_{i\Diamond }$. Then $L'\le 0$ on $\Gamma$ and $L'(X)=0$ if and only if $X=E_{3}.$ By LaSalle’s Invariance Principle, $E_{3}$ is globally asymptotically stable in $\textrm{int}(\Gamma )$. This also implies that $E_{3}$ is unique. The case that $m_{12}^S=m_{21}^S=0$ is similar. $\square$
